# Epithelial stem cells from human small bronchi offer a potential for therapy of idiopathic pulmonary fibrosis

**DOI:** 10.1016/j.ebiom.2024.105538

**Published:** 2025-01-02

**Authors:** Zeyu Liu, Qi Zheng, Zhoubin Li, Moli Huang, Cheng Zhong, Ruize Yu, Rong Jiang, Haotian Dai, Jingyuan Zhang, Xiaohua Gu, Yongle Xu, Chunwei Li, Shan Shan, Feng Xu, Yue Hong, Tao Ren

**Affiliations:** a Department of Respiratory and Clinical Care Medicine, Shanghai Sixth People's Hospital Affiliated to Shanghai Jiao Tong University School of Medicine, Shanghai, 200233, China; b Department of Lung Transplantation and Thoracic Surgery, First Affiliated Hospital, School of Medicine, Zhejiang University, Hangzhou, 310003, Zhejiang, PR China; c Department of Bioinformatics, School of Biological and Basic Medical Sciences, Soochow University, Suzhou, 215123, China; d Key Laboratory of Endocrine Glucose & Lipids Metabolism and Brain Aging, Ministry of Education, Department of Endocrinology, Shandong Provincial Hospital Affiliated to Shandong First Medical University, Jinan, 250021, Shandong, China; e Shandong Engineering Laboratory of Prevention and Control for Endocrine and Metabolic Diseases, Jinan, 250021, Shandong, China; f Department of Otolaryngology, The First Affiliated Hospital, Sun Yat-Sen University, Guangzhou, 510080, China; g School of Life and Health Sciences, Hainan University, Haikou, Hainan 570228, China; h Hainan Province Key Laboratory of One Health, Collaborative Innovation Center of One Health, Hainan University, Haikou, Hainan 570228, China

**Keywords:** Basal stem cells, Human small bronchi, Therapy of idiopathic pulmonary fibrosis, Bronchoscopic implantation, Senescent phenotype

## Abstract

**Background:**

Idiopathic pulmonary fibrosis (IPF) is a fibrosing interstitial pneumonia with restrictive ventilation. Recently, the structural and functional defects of small airways have received attention in the early pathogenesis of IPF. This study aimed to elucidate the characteristics of small airway epithelial dysfunction in patients with IPF and explore novel therapeutic interventions to impede IPF progression by targeting the dysfunctional small airways.

**Methods:**

Airway trees spanning the proximal–distal axis were harvested from control lungs and explanted lungs with end-stage IPF undergoing transplant. Qualified basal cells (BCs, p63/Krt5/ITGA6/NGFR) were expanded, and their cellular functions, feasibility, safety and efficacy for transplantation therapy in IPF were validated with experiments *in vitro* and mouse model. *Single-cell RNA-sequencing* was employed to elucidate the underlying mechanisms governing the BCs based therapy. Based upon these evidences, three patients with advanced IPF and small airway dysfunction received autologous-BCs transplantation. Post-transplantation assessments included lung function, exercise capacity and high resolution computed tomography (HRCT) scans were analyzed to quantify the clinical benefits conferred by the BCs transplantation.

**Findings:**

An overall landscape of senescent phenotype in airway epithelial cells and airway stem/progenitor cells along the proximal–distal axis of the airway tree in IPF were outlined. In contrast to the cells situated in distal airways, BCs located in small bronchi in IPF displayed a non-senescent phenotype, with comparable proliferative, differentiative capabilities, and similar transcriptomic profiles to normal controls. In a mouse model of pulmonary fibrosis, BCs exhibited promising protective efficacy and safety for transplantation therapy. Autologous BCs transplantation in three advanced IPF patients with small airway dysfunction yielded significant clinical improvements in pulmonary function, particularly evidence in lung volume and small airway function.

**Interpretation:**

Epithelia of small bronchi in IPF contain functional and expandable basal stem cells, which exert therapeutic benefits via bronchoscopic implantation. Our findings offer a potential for IPF treatment by targeting small airways.

**Funding:**

10.13039/501100001809National Natural Science Foundation of China (82430001, 81930001, and 81900059), 10.13039/501100014137Shanghai Shenkang Hospital Development Center (SHDC2020CR3063B), 10.13039/100012905Department of Science and Technology of Shandong Province (2024HWYQ-058).

**Research in context**

**Evidence before this study**

Idiopathic pulmonary fibrosis (IPF) is an interstitial lung disease (ILD) that is traditionally characterized by the destruction of alveolar architecture, accompanied by inflammation and fibrosis within the lung parenchyma. Recently, there has been a shift towards an airway-centric perspective in understanding IPF, emphasizing the early pathological features of airway structural alterations and progenitor cell changes in the airway epithelium. This emerging perspective offers new therapeutic directions for IPF management. However, our comprehensive review, which included publications prior to October 2024 encompassing PubMed, Embase, and the Cochrane Library (without restrictions on date or language), revealed no published studies focusing on therapeutic interventions targeting small airways in IPF. The replacement of pathological epithelial cells with functional progenitors has been posited as a promising treatment strategy for fibrotic pulmonary diseases. Nonetheless, the potential of autologous stem cell transplantation has yet to be realized. Currently, there is a lack of published reports characterizing the progenitor cells at different levels of the airways in IPF patients. Moreover, the specific autologous cell types capable of executing regenerative responses, optimized methods for amplifying lung progenitor cells, and the safety of autologous stem cell transplantation require more rigorous exploration. Our study aims to contribute to this field by addressing some of these knowledge gaps.

**Added value of this study**

Our study provides the first overall landscape of senescent phenotype in airway epithelial cells and airway stem/progenitor cells along the proximal–distal axis of the airway tree in IPF, offering novel perspective on the structural intricacies and spatial patterns of epithelial dysfunction. Based on the aging characteristics of epithelial cells at different levels of the airways, we have pioneeringly developed a novel approach to isolate and propagate functional basal cells (BCs). The cultured BCs exhibit stemness and differentiation capabilities comparable to those of healthy (control) BCs and demonstrate favorable safety profiles. Following airway infusion of the expanded autologous tissue stem cells, we observed obvious clinical benefits, including improvements in small airway features, ventilation function and exercise capacity in IPF patients.

**Implications of all the available evidence**

IPF remains a challenging condition with unmet therapeutic needs. Our study proposes a novel cellular therapeutic approach for IPF management, emphasizing the need for further investigation into the specific characteristics of proximal and distal BCs in the progression of IPF. The ability of functional BCs, isolated from the proximal airways, to enhance lung function in IPF patients suggests their potential as an effective treatment option, possibly initiating a paradigm shift in IPF therapy. This study provides the foundational data that support the need for future, larger-scale clinical trials. Additionally, elucidating the molecular mechanisms, particularly those involving PGE2, Wnt, EGFR and FGF signaling pathways, will be critical for optimizing these therapies and extending their applicability to other fibrotic lung diseases.

## Introduction

Idiopathic pulmonary fibrosis (IPF) is a specific form of progressive, fibrosing interstitial pneumonia with poor prognosis, featured with restrictive ventilation and diffusion dysfunction.^1^ Though the explicit mechanism of IPF has not yet been unraveled, accumulating evidence underscores the involvement of alveolar epithelial cell dysfunction during its pathogenesis. The dysfunction encompasses impaired regenerative capacity, cell senescence, and the induction of profibrotic signaling pathways.^2^, ^3^, ^4^ Notwithstanding the application of current anti-fibrotic agents, namely pirfenidone and nintedanib, the therapeutic outcomes in addressing IPF has yet to yield promising results.^1^

Recently, the role of structural and functional defects in the distal airways have gained increasing attention in the pathogenesis of IPF.^5^, ^6^, ^7^ Studies have documented thickened airway walls, distorted airway lumina, loss of terminal bronchioles and ground-glass opacity in radiological assessment in the early-stage of IPF.^6^^,^^7^ Strikingly, these abnormalities often predate the parenchymal fibroproliferative transformations associated with IPF. Current knowledge indicates that conducting bronchioles, around 1 mm in diameter, bifurcate into finer branches, which directly supply the acini and ensure optimal gas exchange efficiency.^8^ These findings engender the hypothesis that small airway obstruction could be either a primary instigator or a synergistic exacerbator of alveolar damages in IPF pathogenesis.^6^^,^^7^ Thus, improving the functions of these bronchioles at early stage of IPF may enhance the supply of fundamental lung units, thereby promoting ventilation and rescuing any loss-of-function acini. Collectively, these findings suggest an airway-centric distribution of IPF.^9^ Restoring the structure and function of injured small airways may provide a strategic therapeutic opportunity to hinder the progression of IPF.

Recently, the role of distal airway epithelial cells in IPF have garnered considerable interest through numerous *single-cell RNA-sequencing* (*scRNA-seq*) studies.^2^^,^^10^, ^11^, ^12^ The senescence of airway stem/progenitor cell subpopulations has been identified as a critical pathogenic factor in IPF progression.^2^ Basal cells (BCs) are essential airway stem cells distributed along the trachea through bronchioles, classically identified by expression of Krt5, p63, ITGA6 and NGFR. These cells are crucial for maintaining homeostasis and airway epithelium regeneration.^4^^,^^6^^,^^13^, ^14^, ^15^ In response to injury, BCs are indispensable to restore the structure and function of impaired airways by virtue of their self-renew and differentiation capacities.^16^ The declines in BCs function may lead to diverse lung diseases, including chronic obstructive pulmonary disease (COPD), obliterative bronchiolitis (OB), and cystic fibrosis (CF).^17^, ^18^, ^19^, ^20^ Therefore, identifying senescent phenotype of BCs across various airway segments and screening for functional BCs have the potential to pave novel therapeutic pathways for IPF treatment.^13^^,^^14^^,^^21^^,^^22^

In this study, we initially delineated an overall landscape of senescent phenotype in airway epithelial cells and airway stem/progenitor cells along the proximal–distal axis of the airway tree in IPF. In comparison to distal airways, BCs (Krt5**+/**p63**+/**ITGA6**+/**NGFR**+**) located in small bronchi in IPF exhibited a non-senescent phenotype. This characteristic suggests their potential for repairing distal airway epithelium. Subsequently, we undertook a meticulous characterization of these BCs, elucidating their cellular functions, feasibility and safety for transplantation therapy. In our pilot clinical trial (ChiCTR2000036648), three individuals with advanced IPF and small airway dysfunction received autologous-BCs transplantation through bronchoscopy and all experienced obvious clinical benefits. This study highlights a fact that the epithelia of small bronchi in IPF patients contain functional and expandable basal stem cells. The therapeutic effects observed following bronchoscopic implantation of these cells indicate a novel strategy to impede IPF progression.

## Methods

### Ethics statement

All procedures performed in studies involving human and animal were conducted in accordance with the ethical standards of the responsible committees and complied with relevant guidelines and regulations.

For the human studies, ethics approval was obtained from Research Ethics Committees of Shanghai Sixth People's Hospital Affiliated to Shanghai Jiao Tong University School of Medicine (Ethics Approval No. 2020-152-(2)) and the First Affiliated Hospital, Zhejiang University School of Medicine (Institutional Review Board approval no. 2021/330). All participants provided informed consent prior to their participation.

For the pre-clinical studies involving mice, ethical approval was obtained from Ethics Committee of Shanghai Sixth People's Hospital Affiliated to Shanghai Jiao Tong University School of Medicine (Ethics Approval No. 2018-0078). All animal experiments were conducted in compliance with IACUC guidelines set forth for the care and use of laboratory animals and approved by the IACUC committee of Shanghai Sixth People's Hospital Affiliated to Shanghai Jiao Tong University School of Medicine.

### Study design

**Fig. 1:**
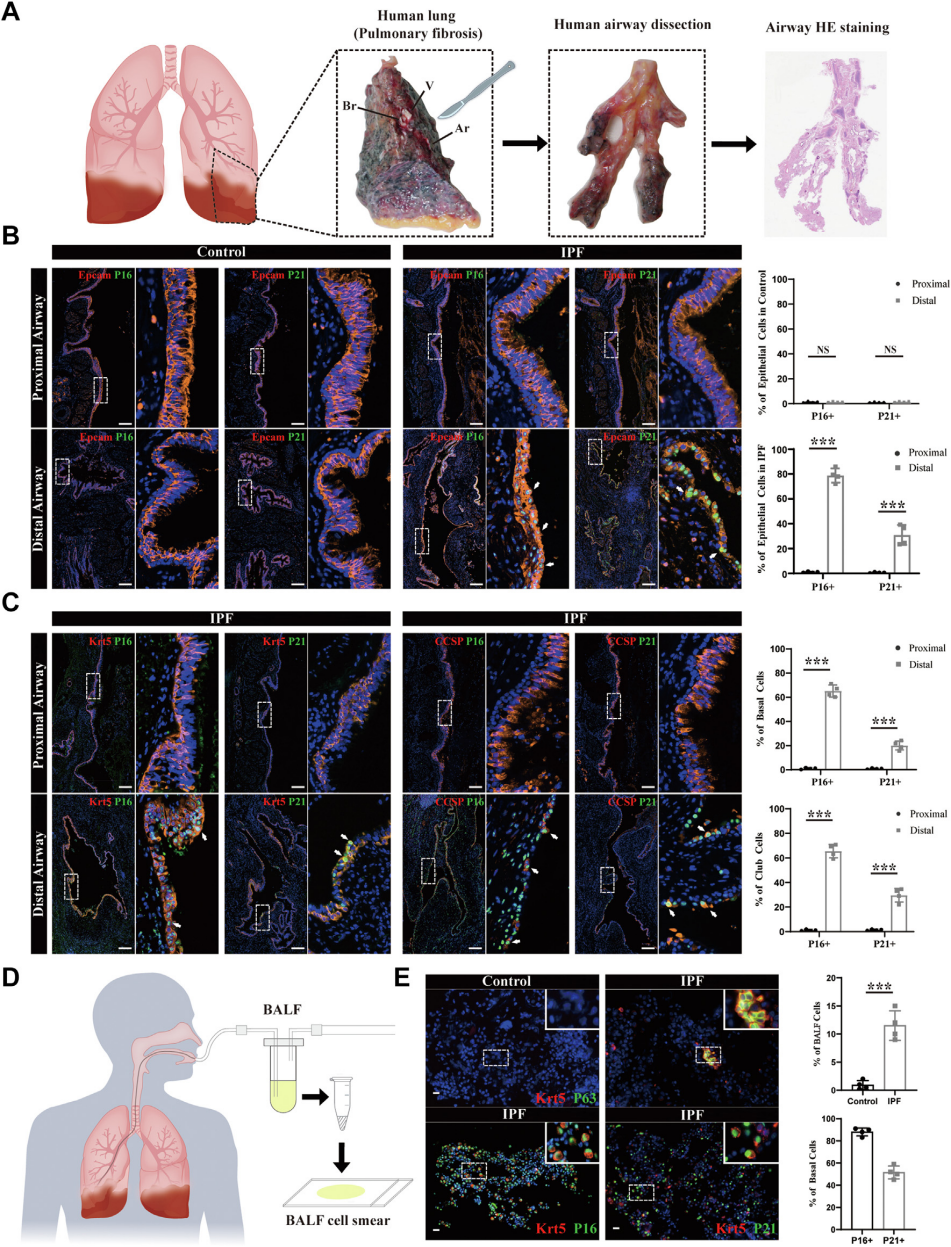
**Landscape of senescent phenotype along the proximal–distal axis of airways in IPF and controls**. **(A)** A schematic diagram illustrating the workflow of airway dissection along the proximal–distal axis. **(B)** Identification of senescence markers p16 and p21 along airway epithelia from control donor lungs (N = 4) and explanted lung lobes (N = 4) with IPF by immunofluorescence staining. Scale bar, 100 μm. The expression ratios of senescence markers in proximal and distal airway epithelia were quantified. **(C)** Distribution of senescent cells in the proximal and distal airways from explanted lung lobes with IPF by immunofluorescence staining. Scale bar, 100 μm. The expression ratios of senescence markers in basal cells and club cells of proximal and distal airways were quantified. **(D)** A workflow of obtaining BALF cells and performing BALF cell smear. **(E)** The proportions of BCs and the expression levels of senescence markers p16 and p21 in BCs within BALF obtained from both control individuals and IPF patients. Scale bar, 20 μm. Data are presented as mean ± SD. ∗∗∗ indicates P < 0.001, while NS denotes no statistical significance (P > 0.05). After Shapiro–Wilk test for normality, two-tailed Student's t-tests were used for panels B, C, and E.

**Fig. 2:**
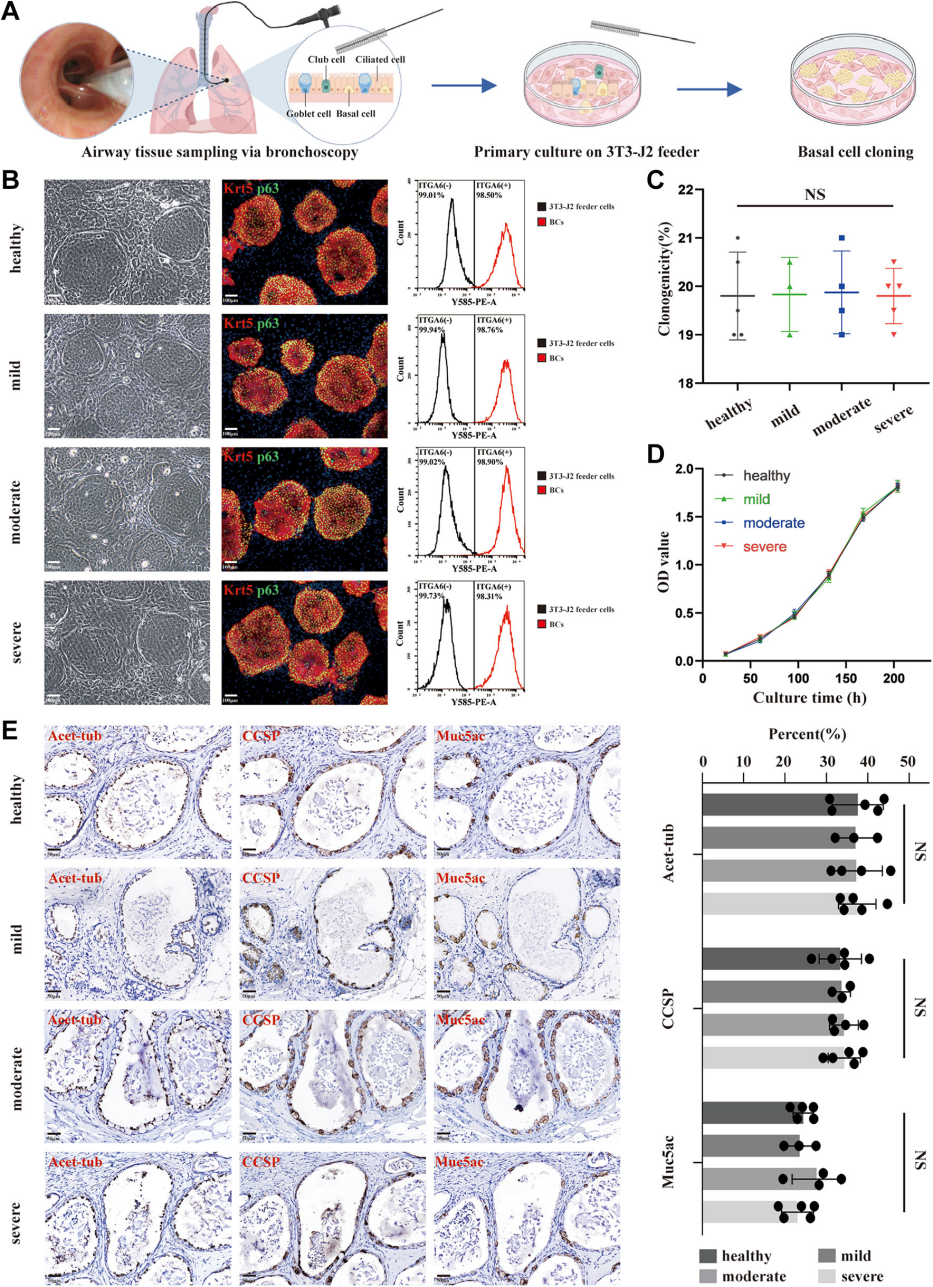
**Isolation and characterization of BCs in mild to severe IPF patients and controls**. **(A)** A schematic diagram illustrating the workflow of BCs isolation. **(B)** Identification of cell characteristics and purity among expanded cultures by flow cytometric analysis for ITGA6 and immunofluorescence staining for Krt 5 and p63. Scale bar, 100 μm. **(C)** Clonogenicity of BCs (number of BCs colonies/number of input BCs). **(D)** Growth curve of BCs. **(E)***In vitro* differentiation of BCs. Major functional cell types were examined by IHC staining (Ciliated cell, Acet-tub; Club cell, CCSP; Goblet cell, Muc5ac). Scale bar, 50 μm. Histogram showed the statistics for the proportions of differentiated cells mentioned above. control, N = 5; mild IPF, N = 3; moderate IPF, N = 4; severe IPF, N = 5. Results are represented as mean ± SD, NS indicated no statistical significance determined by one-way ANOVA after Shapiro–Wilk test for normality (P > 0.05).

This study aimed to identify a novel therapeutic strategy for IPF. We verified the rare occasions of senescence biomarkers within proximal airway epithelium of IPF according to the landscape of senescent phenotype along the proximal–distal axis of airways in surgically resected lung tissue specimens from both IPF (N = 4) and control samples (N = 4). These findings suggested a latent capacity of proximal airway epithelial cells to serve as a reservoir of functional airway stem cells. To further explore this potential, we isolated functional BCs from the fifth-generation bronchi of explanted lungs using bronchial brushing, followed by *in vitro* expansion and culture. To extend our findings towards clinical applicability, we also acquired and expanded proximal airway BCs from IPF patients with varying severity (classified by FVC^23^; mild-IPF (N = 3); moderate-IPF (N = 4); severe-IPF (N = 5)). Additionally, we assessed the functionality of human BCs from archived bronchoscopic brushings in both cell and murine models of fibrosis. Subsequently, we confirmed its genomic stability via whole exome DNA sequencing, and explored the transcriptomic signatures and cellular constitutions using single-cell sequencing analysis. The preclinical safety assessment based on major organ toxicity and tumorigenicity was performed in mice. In light of these points, we identified functional BCs that were capable of compensating for impaired distal airways and could serve as candidates for cellular therapy. Each patient providing samples was diagnosed with IPF according to ATS/ERS/JRS/ALAT guidelines (2018).^24^ The control lung tissue specimens for histologic examination were obtained from non-lesional lung segments of patients undergoing lobectomy for pulmonary nodules, while controls for *in vivo* studies were individuals undergoing bronchoscopy for routine physical examination (all controls had normal pulmonary function and did not have any airway or interstitial lung diseases). Fig. 1 depicts excised lung tissue (*ex vivo* lung), while Fig. 2 illustrates *in vivo* bronchoscopic biopsy specimens. For animal experiments, mice of similar age and weight were randomly assigned to experimental groups. To ensure the integrity and objectivity of the research, investigators and data analyzers were blinded to the group assignment for both mouse and human samples. All data were included in analyses except for outliers (>±2 standard deviations (SDs)). The details of sample sizes or biological replicates for each experiment are provided in the figure and table captions.

A prospective, non-blinded, observational clinical trial was conducted to evaluate the efficacy of autologous basal cell infusion in IPF. The study was registered on the Chinese Clinical Trial Registry (ChiCTR2000036648). Based on the expert assessment and previous experience, the main entry criteria for the study were listed as follows: (i) Age 40–80 years old (including the threshold), regardless of gender; (ii) IPF was diagnosed according to ATS/ERS/JRS/ALAT guidelines (2018); (iii) Subjects with progressive ventilation dysfunction, dyspnea and hypoxemia within 6 months before administration. Forced vital capacity (FVC) was 40%–80% of the predicted value at baseline; (iv) Blood biochemical examination should meet the following standards: alanine aminotransferase (ALT) ≤1.5 μln, aspartate aminotransferase (AST) ≤1.5 μln, total bilirubin (TBIL) ≤1.5 μln, direct bilirubin (DBIL) ≤1.5 μln, blood creatinine (CR) ≤1.5 μln; (v) Expected survival ≥12 months; (vi) Subjects who have good compliance, can understand and cooperate with the completion of pulmonary function examination, are willing to take drugs according to the requirements of the protocol and receive follow-up examination on time; (vii) Subjects who voluntarily participated in the trial, understood and signed the informed consent form. Key exclusion criteria: (i) Subjects with complications, such as tumor, acute infection, hyperthyroidism, heart failure, hepatitis, nephritis, or known history of severe systemic diseases and other organ dysfunction; (ii) Have previously received stem cell therapy, or are intolerant to cell therapy, or have taken drugs that may cause or aggravate pulmonary fibrosis (such as amiodarone, bleomycin or methotrexate); (iii) Subjects who cannot tolerate bronchoscopy; (iv) Have a history of psychotropic drug abuse and drug abuse; (v) Human chorionic gonadotrophin in pregnancy or lactation, or screening β (β- HCG) positive, or unable and unwilling to take effective non drug contraceptives during the study period and 6 months after the end of the study; (vi) Other circumstances that the researcher believes are not suitable for entering this test. After screening, three patients with small airway dysfunction were selected for autologous basal cell infusion with their fully informed consent prior to transplantation. More recipients would be recruited in the future.

### Patient enrollment and sample collection

4 explanted lungs from patients with end-stage IPF undergoing transplant and 4 control lungs (Table 1) were enrolled to depict the landscape of senescent phenotype along the proximal–distal axis of airways. 12 patients with a confirmed diagnosis of IPF and 5 controls (Table 2) were enrolled in the preclinical studies. Patients with IPF were classified into three categories in the light of FVC (% predicted): mild (≥75, N = 3), moderate (55–75, N = 4) and severe (<55, N = 5).^23^ Airway epithelium cells (AECs) were collected by gently advancing the bronchoscopic brushings against mucosal near the opening of the left or right basal segment (fifth-generation bronchi). Mucosa tissues in brushing sites appeared normal via autofluorescence bronchoscopy (green fluorescence color for normal bronchi epithelium). The brush head was then cut with sterile scissors and placed in a special storage solution for fresh specimens. Three patients (Table 3) underwent a clinical trial on BCs transplantation.

**Table 1:** Demographics and clinical characteristics of the patients with IPF and control donors.

Patient	C1	C2	C3	C4
Sex	Female	Male	Male	Male
Age, yr	58	59	73	55
Height, cm	157	166	163	178
Weight, kg	70	65	73	87
BMI	28.4	23.6	27.5	27.5
Former smoker	No	Yes	Yes	No
Time since diagnosis of IPF, yr	None	None	None	None
Comorbidities	None	None	Hypertension	None
Severity stage	None	None	None	None
FVC, L	2.80	4.37	2.66	3.74
FVC, % Pred	110	119	84	83
FEV1, L	2.38	3.44	2.07	3.08
FEV1, % Pred	111	117	86	86
TLC, L	5.22	6.6	4.73	5.86
TLC, % Pred	114	107	80	82
DLCO, % Pred	108	84	92	94
FEF75, L/s	1.19	1.25	0.84	1.51
FEF75, % Pred	91	86	83	80
FEF50, L/s	2.99	4.83	3.31	4.44
FEF50, % Pred	84	117	93	95

Patient	P1	P2	P3	P4
Sex	Male	Female	Male	Male
Age, yr	50	48	72	59
Height, cm	167	158	170	155
Weight, kg	63	63	72	50
BMI	22.6	25.2	24.9	20.8
Former smoker	No	No	Yes	No
Time since diagnosis of IPF, yr	2	1	10	0.5
Comorbidities	Hypertension	Diabetes	Hypertension	None
Severity stage	Severe	Severe	Severe	Severe
FVC, L	1.80	0.98	2.01	0.94
FVC, % Pred	46	32.6	52	38
FEV1, L	1.69	0.78	1.79	0.91
FEV1, % Pred	53	32.6	60	47
TLC, L	2.73	1.29	None	None
TLC, % Pred	47	29	None	None
DLCO, % Pred	19	16.6	None	None
FEF75, L/s	1.45	0.25	1.07	0.87
FEF75, % Pred	88	18.3	97.68	91
FEF50, L/s	3.03	0.86	2.63	1.87
FEF50, % Pred	68.4	24.1	63.9	59

**Table 2:** Demographics and clinical characteristics of the subjects that experienced bronchoscopy brush examination.

Patient	IPF-01	IPF-02	IPF-03
Sex	Female	Male	Female
Age, yr	70	56	77
Height, cm	158	176	155
Weight, kg	50	95	46
BMI	20.0	30.7	19.1
Former smoker	No	Yes	No
Time since diagnosis of IPF, yr	1	6	0.33
Comorbidities	None	None	Hypertension
Severity stage	mild	mild	mild
FVC, L	1.64	3.51	2.2
FVC, % Pred	77.5	79.8	100.3
FEV1, L	1.47	3.16	1.86
FEV1, % Pred	84.9	88.9	117.3
TLC, L	3.21	NA	3.44
TLC, % Pred	73.5	NA	78.1
DLCO, % Pred	38	NA	78.8
FEF75, L/s	0.79	1.71	0.83
FEF75, % Pred	80.9	95.2	185.2
FEF50, L/s	3.1	6.67	2.98
FEF50, % Pred	97.4	145.4	106.5

Patient	IPF-04	IPF-05	IPF-06	IPF-07
Sex	Male	Male	Male	Male
Age, yr	75	70	72	70
Height, cm	172	168	170	170
Weight, kg	80	79	78	70
BMI	27.0	28.0	27.0	24.2
Former smoker	No	Yes	Yes	Yes
Time since diagnosis of IPF, yr	4	5	0.5	0.5
Comorbidities	Hypertension	Diabetes	Hypertension	Hypertension
Severity stage	moderate	moderate	moderate	moderate
FVC, L	2.31	2.16	2.17	2.31
FVC, % Pred	65.9	59	55	60.2
FEV1, L	2.11	1.72	2	1.93
FEV1, % Pred	77.1	62	66.3	64.9
TLC, L	4.24	3.31	4.04	4.31
TLC, % Pred	63.6	51	65	69.5
DLCO, % Pred	48.7	34	41.6	70.9
FEF75, L/s	1.08	0.68	1.46	0.88
FEF75, % Pred	90	53	132.9	76.5
FEF50, L/s	4.73	2.09	3.38	1.84
FEF50, % Pred	123.1	53	81.9	44.1

Patient	IPF-08	IPF-09	IPF-10	IPF-11	IPF-12
Sex	Male	Male	Female	Female	Female
Age, yr	56	65	75	68	61
Height, cm	182	168	165	158	158
Weight, kg	87	60	70	70	61
BMI	26.3	21.3	25.7	28.0	24.4
Former smoker	Yes	Yes	No	No	No
Time since diagnosis of IPF, yr	5	2	0.33	6	6
Comorbidities	Hypertension, Diabetes	None	None	Hypertension, Diabetes	None
Severity stage	severe	severe	severe	severe	severe
FVC, L	2.31	1.44	1.3	1.25	1.14
FVC, % Pred	52	41.6	42.1	44	44.9
FEV1, L	1.85	1.41	1.23	1.06	0.95
FEV1, % Pred	52	51	54	50.1	49.4
TLC, L	3.73	NA	1.83	2.41	2.43
TLC, % Pred	50	NA	35.3	52.4	59.9
DLCO, % Pred	36	NA	23.8	21.4	NA
FEF75, L/s	0.68	1.19	0.9	0.26	0.31
FEF75, %Pred	35	87.5	106.5	31.9	39.8
FEF50, L/s	2.24	4.84	3.03	2.87	1.47
FEF50, %Pred	46	120.9	89.7	91.1	49.4

Patient	NonIPF-01	NonIPF-02	NonIPF-03	NonIPF-04	NonIPF-05
Sex	Female	Male	Male	Female	Male
Age, yr	53	75	38	74	37
Height, cm	165	167	161	158	173
Weight, kg	65	62.5	58	60.5	80
BMI	23.9	22.4	22.4	24.2	26.7
Former smoker	No	Yes	No	No	Yes
Time since diagnosis of IPF, yr	NA	NA	NA	NA	NA
Comorbidities	None	None	None	Hypertension	Hypertension
Severity stage	NA	NA	NA	NA	NA
FVC, L	3.01	3.19	3.14	2.29	3.96
FVC, % Pred	91.3	92	86.5	87.7	84
FEV1, L	2.4	2.79	2.9	1.85	3.42
FEV1, % Pred	91.2	106	95.1	97.3	87.6
TLC, L	4.77	5.49	5.25	5.12	5.66
TLC, % Pred	94.3	91.3	99.3	110.7	84
DLCO, %Pred	82.7	90.5	94.8	95.9	96.4
FEF75, L/s	0.97	0.97	2.01	0.77	1.77
FEF75, %Pred	66.6	117.4	118	120.8	79.9
FEF50, L/s	2.6	2.92	4.23	2.16	3.71
FEF50, %Pred	67.5	76	97.7	71.5	73.3

**Table 3:** Baseline characteristics of three patients enrolled in the clinical trial (ChiCTR2000036648).

Patient	IPF-I	IPF-II	IPF-III
Sex	Male	Female	Female
Age, yr	57	70	61
Ethnicity	Han	Han	Han
Height, cm	182	158	158
Weight, kg	88	52	63
BMI	26.6	20.8	25.2
Former smoker	Yes	No	No
Time since diagnosis of IPF, yr	5.5	1.5	6.5
Comorbidities	Hypertension, Diabetes	None	None
Severity stage	severe	severe	severe
FVC, L	2.09	1.39	1.19
FVC, % Pred	42.2	53.3	42.5
FEV1, L	1.76	1.29	0.92
FEV1, % Pred	44.1	62.7	42.6
TLC, L	3.19	NA	2.09
TLC, % Pred	42.4	NA	46
DLCO, % Pred	28.6	NA	39.9
FEF75, L/s	0.47	0.54	0.20
FEF75, % Pred	23.8	54.3	20.2
FEF50, L/s	2.31	1.44	0.95
FEF50, % Pred	48	44.2	28.8

NA (not applicable or not available).

FVC, Forced Vital Capacity; FEV1, Forced Expiratory Volume in 1 Second; TLC, Total Lung Capacity; DLCO, Diffusing Capacity of the Lung for Carbon Monoxide; FEF75, Forced Expiratory Flow at 75% of FVC; FEF50, Forced Expiratory Flow at 50% of FVC.

### BCs isolation and expansion

AECs were isolated from bronchoscopic brushings through rinsing with wash buffer, and then seeded on a lawn of irradiated 3T3-J2 feeders in a 6-well culture plate (3 wells/brushing, approximately 10,000 brush-off cells per well) as previously described.^25^^,^^26^ BCs with robust self-renewal properties were selectively expanded as colonies on feeder layers at 37 °C in a 7.5% CO_2_ atmosphere. When clones reached around 80% confluence, feeders were removed with a quick TrypLE rinse (30s), and purified BCs were then counted and passaged following complete digestion with TrypLE.

Details about the identification of cellular functions, feasibility and safety for transplantation therapy are provided in the Supplementary Files.

### Clinical trial protocol for BCs transplantation

Three patients diagnosed with advanced IPF were enrolled to explore the clinical efficacy of BCs transplantation (Table 3). Latest pulmonary function assessments before enrollment were collected. Their small airways were impaired (FEF75% P < 80%). BCs were collected by bronchoscopy at enrollment as mentioned above. To prepare for clinical transplantation, clonogenic BCs were purified by negative selection using Feeder Removal Microbeads (Miltenyi Biotech) and cultured in a feeder-free, defined serum-free condition withdrawing antibiotics. The cells were cultured to the required amount and assessed for safety and quality before infusion.

The patients received intratracheal-transplantation of autologous BCs in both lower lobes with a dose of 2∗10^6^ cells/kg by fiberoptic bronchoscopy (Baseline). After infusion, the enrolled patients were followed up for 12 weeks (Visit-2) and 24 weeks (Visit-3), as an extra observation (Visit-2 of patient_1 was lost due to the inconvenient traffic caused by COVID-19). For safety concern of airway administration, immediate pulmonary function was detected 24 h after the transplantation (Visit-1).

### HRCT analysis

Airways, lung parenchyma, and lung density were evaluated by Siemens Syngo®.via Pulmo 3D, which can calculate lung volume and HAV (high attenuation value) area of lungs. HAV areas are defined as the percentage of imaged lung volume with attenuation values between −600 and −250 Hounsfield units (HU).^27^ Artificial intelligence tools, 3D slicer,^28^ were utilized to quantitatively assess the changes in lung parameters, including the air and vascular volume in different pulmonary regions.

### Statistical analysis

All statistical analyses were performed using GraphPad Prism 8.3.0. Normality was assessed using the Shapiro-Wilk (SW) test. Data following a normal distribution were analyzed using parametric tests (two-tailed Student’s t-test for comparisons between two groups; one-way ANOVA, one-way repeated ANOVA for comparisons between three or more groups), while non-normally distributed data were analyzed with non-parametric tests. Kaplan-Meier (KM) survival curves were used, with significance determined by the Log-rank test. P < 0.05 was considered statistically significant and ns denoted no statistical significance. Detailed statistical methods are provided in the figure legends. Data in this study are generally presented as mean ± SD.

### Role of funders

The funders had no role in the experiment design, data collection, data analysis, interpretation, manuscript writing or any aspect of this study.

## Results

### Landscape of senescent phenotype along the proximal–distal axis of airways in IPF and controls

Airway trees spanning the proximal–distal axis were harvested from control lungs and explanted lungs with end-stage IPF undergoing transplant (Fig. 1A). Detailed information for the isolated lung tissues is provided in Table 1. Human airways were categorized into proximal and distal airways based on the presence (proximal) or absence (distal) of cartilaginous structures.^29^ Consistent with previous reports,^2^^,^^30^ the cellular senescence markers p16 (CDKN2A) and p21 (CDKN1A) were significantly elevated in distal airway epithelium from IPF patients compared with controls, which were obtained from non-lesional lung segments of patients undergoing lobectomy for pulmonary nodules. However, immunoreactivity for p16 and p21 was seldom detected in the proximal airway epithelium in both IPF and control samples (Fig. 1B). Co-staining of airway tree sections for senescence markers alongside KRT5 or CCSP, which serve as specific markers of airway stem cells BCs or club cells, showed that p16/p21 apparently expressed in BCs or club cells of distal airways in IPF. However, above expression patterns were notably absent in proximal airways (Fig. 1C). In addition, we found BCs, not typically found in bronchoalveolar lavage fluid (BALF) of control individuals, were present in BALF of IPF patients. The substantial proportion of these BCs displayed hallmarks of senescence (Fig. 1D and E). Collectively, these data indicate that BCs residing in distal airways of individuals affected by IPF demonstrated functional impairments, whilst the rare occasions of senescence biomarkers within proximal airway epithelium of IPF suggested a latent capacity for serving as a reservoir of functional airway stem cells. Subsequently, we innovatively designed a bronchial brushing sampling method for cultivation. The technology successfully isolates and expands non-senescent airway BCs from *ex vivo* specimens (details in Figure S1).

### Characterization of small bronchi–derived BCs in IPF and controls

To enhance the clinical applicability of BCs, we expanded our sampling techniques to *in vivo* studies. Through this approach, we acquired BCs from small bronchi-derived in mild to severe stages of IPF (mild IPF, N = 3; moderate IPF, N = 4; severe IPF, N = 5) and controls (N = 5) (Fig. 2A). Basic information of enrolled participants was shown in Table 2. The cellular functions of BCs were then comprehensively characterized and identified. Through marker staining and flow cytometry analysis, clones from IPF (mild, moderate, severe) and control groups showed no detectable difference in immunoreactivity to BCs markers Krt5 and p63, and reached comparable purities of 98% or above based on ITGA6 expression (Fig. 2B). In aspects of proliferation and differentiation capability, BCs obtained from patients with various severity of IPF displayed a similar proliferation ability compared to controls in terms of BCs growth curve and clonogenicity (Fig. 2C and D). Differentiation capability of patient-derived BCs was examined by *in vivo* subcutaneous implantation assay. Compared with controls, patient-derived BCs showed the equivalent capability to differentiate into airway major cell types-ciliated, goblet, and club cells (Fig. 2E). Above all, small bronchial BCs in patients with mild to severe IPF exhibited comparable characteristics to those under normal physiology.

### Characterization of BCs across passages

**Fig. 3:**
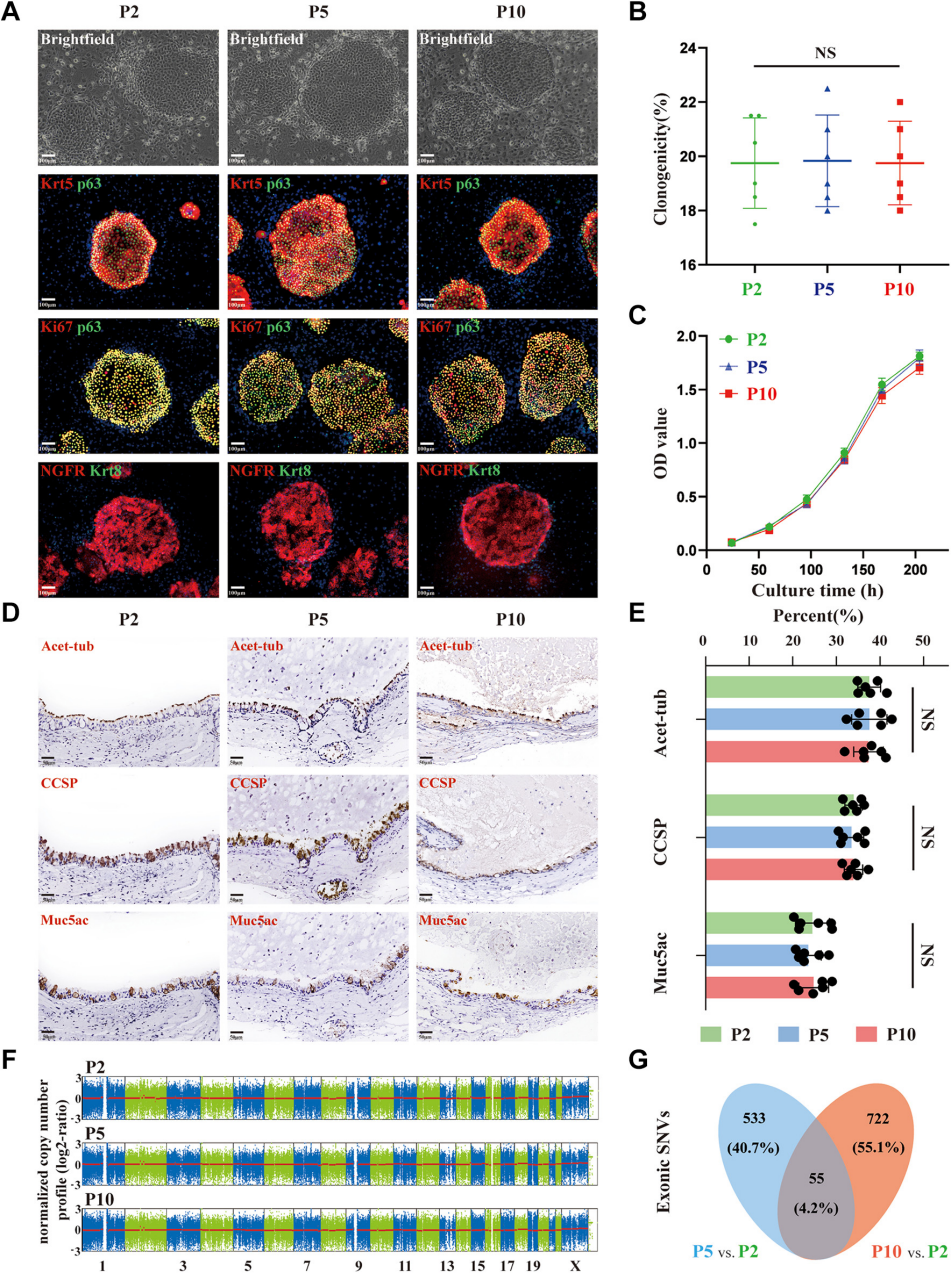
**Characterization of BCs at different passages**. **(A)** Immunofluorescence staining for BCs markers Krt5/p63/NGFR, proliferation marker Ki67, differentiation marker Krt8 in IPF clones across passages. Scale bar, 100 μm. **(B and C)** Clonogenicity (B) and growth curve (C) of IPF-BCs at passage 2, 5 and 10 (number of BCs colonies/number of input BCs). **(D and E)** IHC staining (D) for airway major cell types from *in vitro* differentiation of BCs at different passages, and the summary statistics (E) for proportion of each cell type. Scale bar, 50 μm. **(F)** CNV analysis depended on the whole exome sequencing results of BCs at passage 2, 5 and 10 by using CNVkit. **(G)** Venn diagram showing the number of all detected exonic SNVs (synonymous, non-synonymous, indels) in passage 5 and 10 relative to passage 2. N = 6 in each passage. Results are represented as mean ± SD, NS indicated no statistical significance determined by one-way repeated ANOVA (P > 0.05).

To further assess the stability and fate commitment of IPF patient-derived BCs, we performed characterization and whole exome sequencing studies on BCs from continuous culture propagation. As indicated in Fig. 3A, IPF clones across passages maintained uniformly expression of BCs markers Krt5/p63/NGFR with alike clonal morphology and were positive in proliferation marker Ki67, as well as free of differentiation marker Krt8.^15^ As the number of generations increased, BCs displayed stable self-renewal ability reflected by the similar extent of Ki67 positivity, statistically consistent clonogenicity and cell proliferation rates (Fig. 3B and C). Differentiation capability of BCs at different passages was further examined. The proportion of airway major cell types from *in vitro* differentiation of BCs showed no statistically significant difference (Fig. 3D and E).

Furthermore, the genomic stability of BCs was monitored and confirmed during cell expansion, based on whole exome DNA sequencing. IPF-BCs with increasing passages showed absences of cell fate-associated copy number variations (CNV) (Fig. 3F, Data File S1). When compared with early generation of BCs (Passage 2), 588 and 777 single nucleotide variations (SNVs) events (synonymous, nonsynonymous, indels) were observed in Passage 5 and Passage 10 BCs, whilst only 55 variations concurrent in both passages (Fig. 3G). These number were aligned with random mutations occurred in other somatic cells and within a reasonably narrow range.^31^ In addition, percentage of mutant reads in each variation is quite low over total number of reads, indicating that SNVs only presented in a small fraction of BCs (Data File. S2). To examine potential risks, we inspected the SNVs, especially the co-occurring variations, and cross-referenced them in disease association databases. Functional variant sites contributed to additional major illness such as malignant tumor, severe cardiovascular and cerebrovascular events, were not identified inside BCs, although some mutations were present randomly (Data File. S2). Furthermore, we analyzed the function of genes where these SNVs are commonly located. Adverse events, including gene alterations linked to tumorigenesis, senescence or programmed cell death were not found in BCs by generation ten (Data File S2). Conclusively, these BCs presented robust genomic stability after expansion *in vitro*.

Based on aforementioned investigations, we confirmed that IPF-BCs preserve the functions of airway epithelial stem cells, even after extensive *in vitro* propagation. Further we conducted *scRNA-seq* to assess the genomic characteristics of cultured BCs. The results indicated that the two groups of BCs shared similar genomic features, exhibiting good stemness and biosafety (Figure S2). By analyzing their interactions with distal BCs and fibroblasts, we can infer their therapeutic potential (Figure S3). BCs hold promise as seed cells for cell transplantation therapy in IPF.

### Intra-tracheal transplantation of BCs in bleomycin-injured mice

**Fig. 4:**
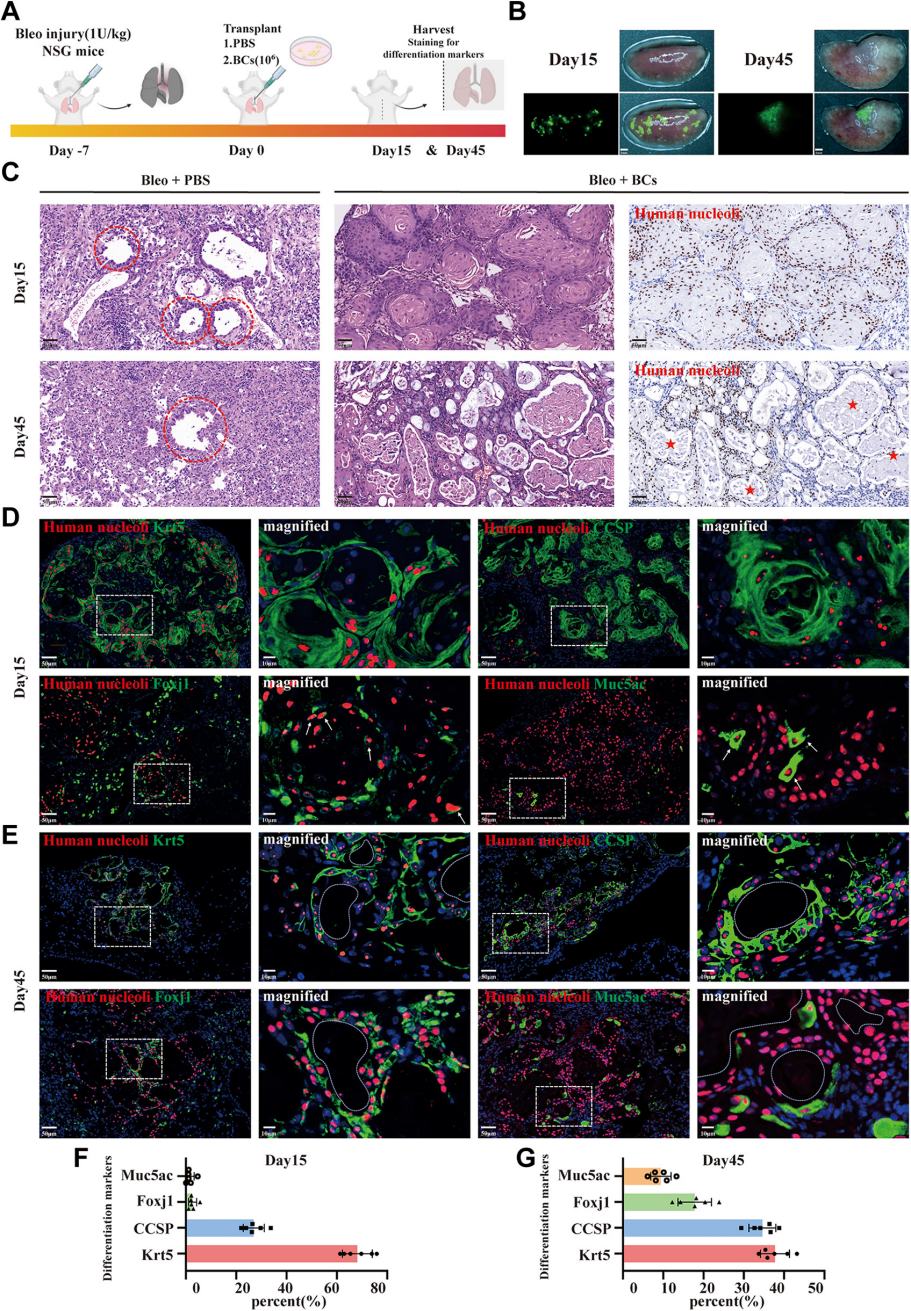
***In vivo* differentiation capacity of transplanted BCs in bleomycin-injured murine lung**. **(A)** Flow chart of experimental procedure. **(B)** Merged images of fluorescent and bright field showing the colonization of transplanted BCs (GFP labeled, green) in injured lung lobes at day 15 and day 45. Scale bar, 2 mm. **(C)** HE staining and IHC staining targeting anti-human nucleoli for histomorphometric analysis of the injured mouse lungs with or without BCs transplantation. Red dashed lines denoted the injured airways in fibrotic regions. Asterisks indicated the newly formed lumen structures. Scale bar, 50 μm. **(D and E)** Representative fluorescence micrographs demonstrating the differentiation conditions of engrafted BCs at day 15 (D) and day 45 (E). Human nucleoli, red; Krt5, green; CCSP, green; Foxj1, green; Muc5ac, green. Arrowheads indicate examples of double-stained cells. White dashed lines outline lumen structures. Scale bar, 20 μm. **(F and G)** Summary statistics for the proportions of airway major cell types from *in vivo* differentiation of IPF-BCs at day 15 (F) and day 45 (G). Basal cells, Krt5; Ciliated cell, Foxj1; Club cell, CCSP; Goblet cell, Muc5ac. Each group consisted of 6 mice.

To assess BCs functions and protective potential *in vivo*, we transplanted patient-derived BCs by intratracheal instillation in mice with bleomycin-induced pulmonary fibrosis, which is probably the most broadly used model to study lung fibrogenesis.^9^^,^^32^, ^33^, ^34^, ^35^ The timeline for the treatment procedure and sample collection was illustrated in Fig. 4A. As Fig. 4B shown, GFP-labelled BCs were able to move towards and settle in injured mouse lobes. Histopathologic analysis revealed that airway epithelial structure within areas of fibrosis appeared less intact after bleomycin injury, consistent with previous reports.^36^, ^37^, ^38^ Moreover, colonized BCs progressively repaired the damaged areas, and even formed airway lumen structures over time (Fig. 4C). Subsequently, we tracked the dynamic changes of engrafted human BCs by staining of specific differentiation markers. The majority of colonized BCs maintained a Krt5+ phenotype and exhibited robust self-renewal capacity at 15-day post intratracheal implantation. After 45 days, these cells progressively differentiated into polarized lumens covered by mainly club, ciliated cells, and occasional Muc5ac-expressing cells (Fig. 4D and E, Figure S4). The proportion of the major airway cell types from *in vivo* differentiation of IPF-BCs were quantitatively assessed over time (Fig. 4F and G). Consequently, we confirmed that the *in vitro* differentiation capacity of BCs is preserved in an *in vivo* setting. These observations support the hypothesis that BCs from small bronchi may serve as an airway modulator and restorer for treatment of IPF, by addressing the pathological features of reduced and dysfunctional small airway.

**Fig. 5:**
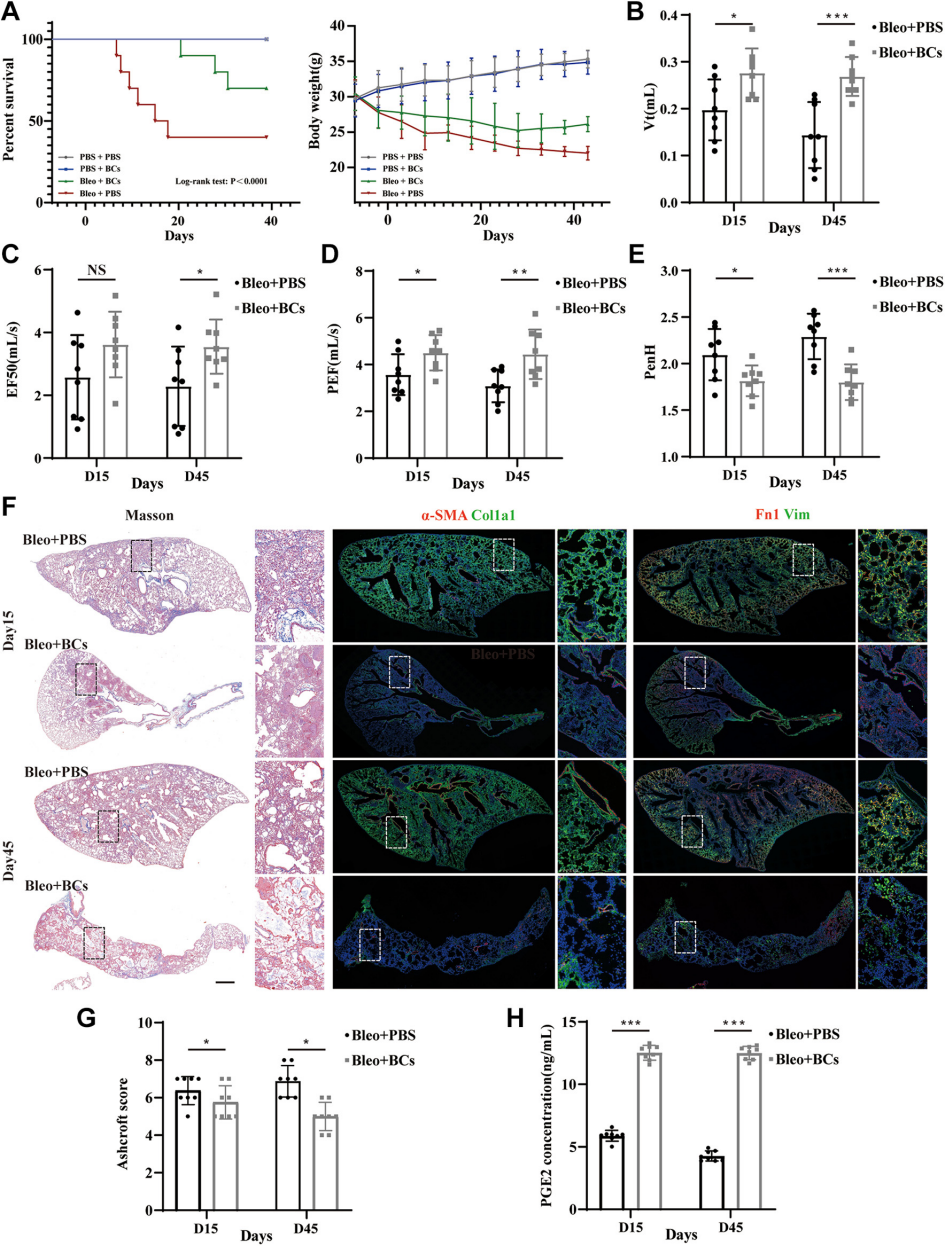
**Functional BCs infusion in bleomycin-injured murine lung exerts potential protectiveefficacy**. **(A)** The survival time and body weight of mice with or without bleomycin treatment and BCs transplantation. Each group contained 20 mice. **(B**–**E)** Whole-body plethysmography (WBP) measurements were conducted to assess lung function, including volume indicators such as tidal volume (Vt); airway function indicators including expiratory flow rate at 50% of exhaled volume (EF50, ml/s) and maximum expiratory flow rate (PEF, ml/s); enhanced pause (PenH). (N = 8 mice per group) **(F)** Masson staining and immunofluorescence (IF) staining of the injured mouse lungs with or without BCs transplantation at day 15 and day 45. Collagen fibers are shown in blue (Left). Fibrosis-associated markers, α-smooth muscle actin (α-SMA) are shown in red, collagen 1a1 (Col1a1) are shown in green (Medium). Fibronectin (FN-1) are shown in red, Vimentin (VIM) are shown in green (Right). (N = 8 mice per group, scare bar = 400 μm). **(G)** Ashcroft scores of the injured mouse lungs with or without BCs transplantation at day 15 and day 45. (N = 8 mice per group) **(H)** PGE2 concentration in bronchoalveolar lavage fluid (BALF) obtained from the injured mouse lungs with or without BCs transplantation at day 15 and day 45. BALF was collected by bronchoalveolar lavage using 1 ml PBS. (N = 8 mice per group). Results are represented as mean ± SD. ∗P < 0.05, ∗∗∗P < 0.001. Statistical analyses were performed using the Log-rank test for survival analysis (A). Two-tailed student's t-test were conducted in panel (B–E, G–H).

Of particular note, the application of BCs in bleomycin-injury mouse model led to a substantial improvement in both survival time and body weight (Fig. 5A). Furthermore, pulmonary function tests were conducted on post-transplantation mice. The results demonstrated significant improvements in pulmonary function indicators, including tidal volume (Vt); enhanced pause (PenH)^39^; and airway function indicators such as expiratory flow rate at 50% of exhaled volume (EF50, ml/s), maximum expiratory flow rate (PEF, ml/s). A single infusion of BCs significantly curtailed the progression of fibrosis after lung injury (Fig. 5F and G). We find a remarkable reduction in the differentiation of fibroblasts (fibrosis-associated markers, such as α-SMA, Col1a1, Vimentin and Fibronectin) in mice that received BCs transplantation treatment. Notably, the degree of fibrosis was significantly diminished, particularly in areas adjacent to the engrafted cells (Figure S5), which may rely on Prostaglandin E2 (PGE2) secreted by transplanted BCs (Fig. 5H). Accordingly, we speculate that the transplantation of functional BCs may serve as a protective factor to prevent the development of fibrosis. The safety assessment of BCs transplantation was also evaluated (Figures S6 and S7).

### Clinical efficacy of autologous BCs transplantation in IPF patients

**Fig. 6:**
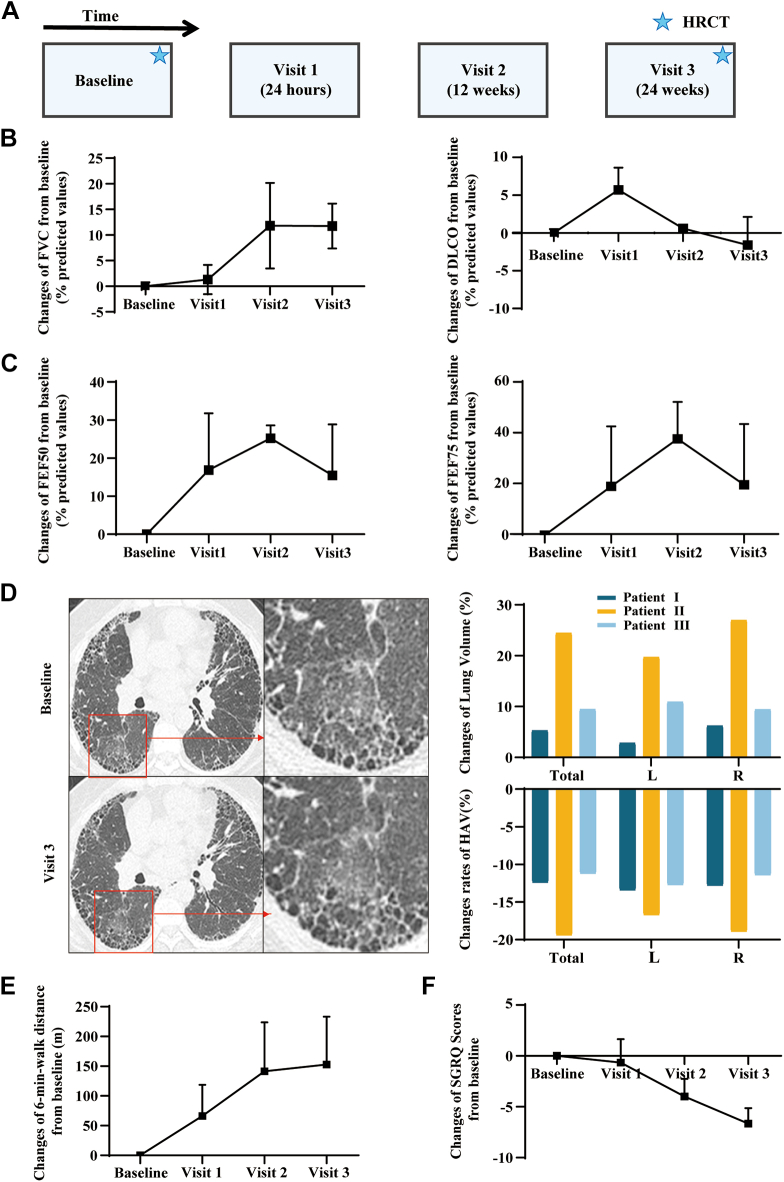
**Clinical trial data for autologous BCs transplantation in IPF patients**. **(A)** A timeline illustrating the design of the clinical trial. **(B and C)** Line chart showing the trend change of FVC (% of predicted), DLCO (% of predicted), FEF 50 (% of predicted) and FEF 75 (% of predicted) during the follow-up period in three patients. **(D)** Chest HRCT images and the ground-glass opacity zoom-in of the identical level in three patients at baseline and visit 3. Density data from Syngo®.via was labeled in bottom-right. Histograms depicting change rates of lung volume and HAV area. L denotes left, R right. **(E** and **F)** Line chart showing the trend change of 6MWT distance and SGRQ Scores during the follow-up period in three patients, respectively.

Three IPF patients underwent autologous BCs transplantation based on establish protocols in our clinical trial (Fig. 6A). After BCs infusion, a reversed pulmonary function was noted in these subjects. FVC increased continuously in all patients during post-treatment follow-up period. At week 24, FVC showed a significant increase compared with baseline, from 42.2%p to 50.5%p, 53.3%p to 70%p and 42.5%p to 52.7%p in patient_1, patient_2 and patient_3, respectively (Fig. 6B). DLCO rapidly ascended 24 h after transplantation and kept stable following a slight decline (Fig. 6B). As a representation of small airway functions, FEF75 and FEF50 greatly improved 24 h after transplantation. The overall improvement in these parameters was observed across all patients, albeit with varying trajectories (Fig. 6C).

The changes in radiography by HRCT were observed and analyzed from multiple perspectives. An increased lung volume was confirmed by HRCT after transplantation. The whole lung volume at the end-expiratory phase increased 210 ml in patient_1, 535 ml in patient_2 and 137 ml in patient_3 at visit 3. The volume of right and left lungs increased uniformly, though the transplantation was performed at single lung lobes (Fig. 6D). At the same time, the attenuation of lung density was identified by the decline of HU. Decrease of HAV area (the percentage of imaged lung volume with attenuation values between −600 and −250 HU) was also observed in the whole lung, which symbolizes the absorption of inflammation and excess extracellular matrix, the reduction of interstitial fibrosis, and the increases in ventilation (Fig. 6D).^27^

Furthermore, the artificial intelligence-assisted analysis was implemented to assess the changes in different pulmonary regions, including the total lung air and vascular volume. Based on preoperative and postoperative comparisons, all three patients exhibited the notable increase in lung air content. Patient_1 experienced a remarkable 140% rise, Patient_2 had a 15% increase, while Patient_3 showed an 82% augmentation in their lung air volume. The increased lung volume, as depicted in the CT scans, indicates that the intervention has potentially led to improved airway patency and expansion of the lung parenchyma. The correlation between the CT findings and the FVC measurements provides strong evidence for the effectiveness of the treatment in improving the patients' respiratory mechanics. The enhanced lung ventilation, evidenced by the FVC improvement, reflects the increased ability of the lungs to hold and expel air. This is a key indicator of lung function and is closely related to the structural changes observed in the CT scans. In addition, significant increase of vascular volume indicated improved lung parenchyma, which is a positive sign for lung healing and functional recovery. These findings, while variable among patients, provide valuable insights into the therapeutical outcomes and the complex interplay between lung function, and vascular changes following the intervention.

Significant improvements on dyspnea and life qualities were thereupon recognized. All patients felt remission of dyspnea and reduced oxygen requirement. The patients’ SpO_2_ reached 95% or above without oxygen inhalation. Exercise capacity was evaluated by 6MWT, which showed longer distances with an average increase of 153 m (Fig. 6E). Overall amelioration was confirmed by the reduction of SGRQ scores (Fig. 6F). These improvement effects were kept for months, and better life qualities were reported during the follow-up period. No additional side effects were noticed.

Collectively, autologous BCs transplantation in IPF patients showed a significant clinical efficacy, especially improvement in lung volume and small airway function.

## Discussion

This is the inaugural comprehensive investigation of senescent phenotype along the proximal–distal axis of airways in IPF, providing novel perspective on the structural intricacies and spatial patterns of epithelial dysfunction. Impaired function of distal airway epithelial cells hinders the reparative processes of small airways, then potentially indicative of a graver outcome in IPF.^10^^,^^30^^,^^40^^,^^41^ The rare occasions of senescence biomarkers within proximal airway epithelium suggested a latent capacity for serving as a reservoir of functional airway stem cells. Pioneeringly, we isolated and propagated non-senescent BCs (Krt5+/p63+/ITGA6+/NGFR+) from small bronchi, and the following up cultures exhibited comparable airway stem cell function compared to healthy counterparts, notably in terms of self-renew capacity and lineage commitment. Intratracheal transplantation of BCs in mice demonstrated that BCs cultures contributed to reconstituting the injured epithelium in fibrotic regions, accompanied by a favorable safety profile. Subsequently, three patients with severe IPF in a state of disease progression proceeded to receive a single airway infusion of autologous BCs via bronchoscopy, followed by a 6-month clinical evaluation. As anticipated, all patients displayed marked therapeutic responses, manifesting as improvements in small airway features and ventilation function. Collectively, our findings provided strong evidence that BCs infusion played critical roles in modulating, and potentially reversing, the impaired function of small airway in IPF. This highlights their potential as a compelling therapeutic avenue for IPF management.

In addition, the substantial improvement of FVC in these 3 patients was impressive in line with the small airway function improvement. The small airway is the core structure of the secondary lung lobules,^8^ and its destruction will not be conducive to the free flow of air into and out of the alveoli, thus accelerating the alveoli collapse. This is aligned with an idea that changes in the distal conducting airways potentially enhance injury or disrupt repair responses in alveoli.^42^ Thus, we hypothesize that the secondary lung lobules are involved in early IPF due to hypoventilation and the improvement in small airways may help with refilling of collapsed alveoli. Furthermore, the repaired distal airway epithelium might consist of epithelial progenitors or provide a niche to regulate alveolar regeneration.^43^ Based on the inferences above, structural and functional recovery of small airways leads to the improvement in FVC.

In view of previous clinical researches, steady rates of FVC reduction have been reported in patients with IPF, ranging from 100 to 200 ml loss of FVC per year for patients under anti-fibrotic treatments to 200–400 ml loss per year for those without medications.^1^ Over the past decade, researchers worldwide have made endeavors to treat IPF by cell-based therapies with attempts at using mesenchymal stem cells and alveolar type II cells.^44^, ^45^, ^46^, ^47^, ^48^ To date, the most striking outcome has been reported by bone marrow MSCs transplantation, which managed to raise FVC% predicted by 7.8%.^44^ Our clinical observations herein show that BCs infusion is able to further prevent deterioration and reverse the declining trend of lung function parameters. This is a preliminary indication that airway epithelial stem cell-based therapy has a great potential to reverse impaired pulmonary function of IPF.

Notably, the pulmonary function of these three subjects has immediate post-treatment improvement. This suggests that there might be some regulatory factors participating in the rapid response. Club cell secretory protein (CCSP, also called SCGB1A1) is an anti-inflammatory protein secreted by epithelial cells and can attenuate airway mucus production.^49^ Loss of club cells (CCSP-marked cells) correlates with bronchiectasis and fibrotic changes in ILD.^50^ Replenishing CCSP-expressing cells via BCs supplementation is likely to be one of the advantageous factors contributing to symptom improvement. PGE2 is another immunosuppression mediators secreted by epithelia cells. It is involved in diverse physiological processes including immune regulation, angiogenesis and reversal of bronchoconstriction.^51^^,^^52^ In our study, we confirmed that some BCs expressed PGE synthases (PTGES) could further secret PGE2, which may partially explain the rapid regulation of pulmonary function. Moreover, PGE2 is implicated in the activation of endogenous stem cells, which is instrumental for the modulation of tissue repair, promotion of angiogenesis and anti-fibrotic effect by interacting with adjacent fibroblasts.^51^, ^52^, ^53^ Thus, PGE2 could enhance the overall regenerative capacity of the lung tissue, thereby supporting the sustained benefits of BC-based therapies.

This study has several limitations. First, the protective effects of BCs transplantation may be mediated by the interaction between infused BCs and local distal BCs or fibroblasts. In addition to the PGE2, Wnt, FGF and EGF activation reported in this project, further investigations are needed to verify the potentially complex mechanisms involved in these interactions and their impact on lung function. Second, bleomycin-induced pulmonary fibrosis mice model was utilized in this study. Considering the fact that the cause of IPF is still unclear, the bleomycin-induced pulmonary fibrosis in mouse and the idiopathic pulmonary fibrosis in human might be different. Although murine models provide valuable insights into disease mechanisms and protective interventions, their relevance to the human condition remains uncertain due to the inherent limitations imposed by the lack of a true IPF animal model. Third, given the challenges of recruiting qualified patients with advanced IPF, sample sizes are not big. Despite these constraints, we believe that our findings offer meaningful contributions to the IPF management and set the stage for future research with larger cohorts. It is our hope that this work will encourage further investigation and facilitate the recruitment of a larger eligible patient population in subsequent studies to validate and expand upon our results.

In conclusion, we documented BCs from human small bronchi as a novel potential IPF treatment. Through rigorous validation of their cellular functionality, coupled with evidence from autologous BCs transplantation in IPF patients, we demonstrate their therapeutic efficacy in ameliorating small airway function and lung volume. These findings not only illuminate the significant role of small airways in the pathogenesis of IPF, but also pave the way for innovative and targeted treatment approaches. By focusing on the enhancement of small airway function, BCs based treatment could address a critical aspect of IPF that have not been explored before. We acknowledge that further exploration to optimize BC-based therapies and its combination with existing treatment is needed. The preliminary success and the mechanistic insights provided by our study highlight the transformative potential to revolutionize IPF management.

## Contributors

All authors had full access to all of the data in the study. T.R., Y.H., S.S., F.X., and Z.-Y. L. designed the project and took responsibility for the integrity of the data and the accuracy of the data analysis. Z.-Y. L. performed most of the experimental work, data analysis and wrote the original draft. Z.-B. L. provided the clinical samples. M.-L. H. and R.J. assisted with the bioinformatic analysis. C.Z. participated in the animal experiments. T.R., F.X., Q.Z., R.-Z. Y., H.-T. D., X.-H. G., and Y.-L. X. performed the clinical experiments and analyzed the data. C.W.-L. constructed GFP plasmids. T.R., Y.H., S.S., F.X. and J.-Y. Z. reviewed the manuscript and provided helpful comments.

## Data sharing statement

All the data and materials involved in this study are present in the paper or the Supplementary Materials. The raw sequencing data and processed data included in this study have been deposited in the Gene Expression Omnibus (GEO) database with the accession number GSE282149.

## Declaration of interests

The authors declare no competing interests.

## Appendix A Supplementary data

Supplementary Materials

Graphical Abstract
