# Supplementary material for: Epithelial stem cells from human small bronchi offer a potential for therapy of idiopathic pulmonary fibrosis
Source: eBioMedicine. 2025 Jan 2;112:105538. doi: 10.1016/j.ebiom.2024.105538 (PMC11754162; doi:10.1016/j.ebiom.2024.105538)
Supplement: Data File S3 [file mmc3.pdf]

	Segment	Value before op(cm^3)	Value after op(cm^3)	Difference(cm^3)	Change Rate
IPF-I	Vessels right	13.0631	65.0815	52.0184	3.982086947
	Vessels left	8.65705	47.8862	39.22915	4.531468572
	Vessels left ventral	5.22024	24.6522	19.43196	3.722426555
	Vessels left dorsal	3.43681	23.234	19.79719	5.760338803
	Vessels left upper half	3.1283	18.1316	15.0033	4.795991433
	Vessels left lower half	5.52876	29.7546	24.22584	4.381785427
	Vessels left upper	1.31162	7.34768	6.03606	4.601988381
	Vessels left middle	5.1106	23.9629	18.8523	3.688862364
	Vessels left lower	2.23483	16.5756	14.34077	6.416939991
	Vessels right ventral	5.13743	29.8671	24.72967	4.813626658
	Vessels right dorsal	7.92572	35.2144	27.28868	3.443053754
	Vessels right upper half	4.33786	25.5145	21.17664	4.881817302
	Vessels right lower half	8.72529	39.567	30.84171	3.534748988
	Vessels right upper	1.53149	9.64252	8.11103	5.29616909
	Vessels right middle	8.51534	36.5145	27.99916	3.288084798
	Vessels right lower	3.01632	18.9244	15.90808	5.274002758
	Vessels right upper lobe	4.3851	26.0571	21.672	4.9421906
	Vessels right middle lobe	1.41427	8.83089	7.41662	5.244133016
	Vessels right lower lobe	7.26378	30.1935	22.92972	3.156720055
	Vessels left upper lobe	3.63043	21.3737	17.74327	4.887374223
	Vessels left lower lobe	5.02662	26.5125	21.48588	4.274418993
	Total lung air volume	71.097	170.368	99.271	1.396275511
IPF-II	Vessels right	11.1194	57.2446	46.1252	4.148173463
	Vessels left	6.99735	40.3116	33.31425	4.760980943
	Vessels left ventral	2.57707	14.7025	12.12543	4.705122484
	Vessels left dorsal	4.42028	25.6091	21.18882	4.793547015
	Vessels left upper half	2.03633	16.7603	14.72397	7.230640417
	Vessels left lower half	4.96101	23.5514	18.59039	3.747299441
	Vessels left upper	0.856161	8.17275	7.316589	8.545809725

	Vessels left middle	3.9525	16.8627	12.9102	3.266337761
	Vessels left lower	2.18868	15.2762	13.08752	5.979640697
	Vessels right ventral	2.72727	16.0561	13.32883	4.887242554
	Vessels right dorsal	8.3921	41.1885	32.7964	3.908008722
	Vessels right upper half	4.6134	25.6689	21.0555	4.563987515
	Vessels right lower half	6.50597	31.5758	25.06983	3.853357762
	Vessels right upper	2.29812	14.2496	11.95148	5.200546534
	Vessels right middle	6.82354	27.903	21.07946	3.089226413
	Vessels right lower	1.99771	15.092	13.09429	6.554650074
	Vessels right upper lobe	3.18432	17.7088	14.52448	4.561250126
	Vessels right middle lobe	1.0922	7.89164	6.79944	6.225453214
	Vessels right lower lobe	6.84285	31.6442	24.80135	3.624418188
	Vessels left upper lobe	2.28953	16.3427	14.05317	6.138015226
	Vessels left lower lobe	4.70781	23.9689	19.26109	4.091305724
	Total lung air volume	38.9886	45.0303	6.0417	0.154960681
IPF-III	Vessels right	38.5322	23.7333	-14.7989	-0.384065794
	Vessels left	30.7784	17.085	-13.6934	-0.444902919
	Vessels left ventral	6.11314	4.0085	-2.10464	-0.344281335
	Vessels left dorsal	24.6652	13.0765	-11.5887	-0.469840099
	Vessels left upper half	10.2314	6.77165	-3.45975	-0.338150204
	Vessels left lower half	20.5469	10.3133	-10.2336	-0.498060535
	Vessels left upper	3.49637	2.20753	-1.28884	-0.368622314
	Vessels left middle	13.0205	9.31143	-3.70907	-0.284863869
	Vessels left lower	14.2615	5.56602	-8.69548	-0.60971707
	Vessels right ventral	8.03266	5.56228	-2.47038	-0.30754196
	Vessels right dorsal	30.4996	18.171	-12.3286	-0.404221695
	Vessels right upper half	9.13411	7.07399	-2.06012	-0.225541405
	Vessels right lower half	29.3981	16.6593	-12.7388	-0.433320521
	Vessels right upper	2.97228	1.95659	-1.01569	-0.341720834
	Vessels right middle	14.545	11.7222	-2.8228	-0.194073565
	Vessels right lower	21.015	10.0544	-10.9606	-0.52156079

	Vessels right upper lobe	13.6277	10.6059	-3.0218	-0.221739545
	Vessels right middle lobe	1.79733	1.45985	-0.33748	-0.187767411
	Vessels right lower lobe	23.1072	11.6676	-11.4396	-0.495066473
	Vessels left upper lobe	12.5556	17.085	4.5294	0.360747396
	Vessels left lower lobe	18.2228	0	-18.2228	-1
	Total lung air volume	64.3756	117.074	52.6984	0.818608293
