# Supplementary material for: Epithelial stem cells from human small bronchi offer a potential for therapy of idiopathic pulmonary fibrosis
Source: eBioMedicine. 2025 Jan 2;112:105538. doi: 10.1016/j.ebiom.2024.105538 (PMC11754162; doi:10.1016/j.ebiom.2024.105538)
Supplement: Supplementary Materials [file mmc4.docx]

Supplementary Materials for

**Epithelial stem cells from human small bronchi offer a potential for therapy of Idiopathic Pulmonary Fibrosis**

Zeyu Liu, Qi Zheng, Zhoubin Li, Moli Huang, Cheng Zhong, Ruize Yu, Rong Jiang, Haotian Dai, Jingyuan Zhang, Xiaohua Gu, Yongle Xu, Chunwei Li, Shan Shan*, Feng Xu*, Yue Hong*, Tao Ren*.

*Corresponding author. Email: liuyuanrentao@sjtu.edu.cn (T.R.); yuehongsls@hainanu.edu.cn (Y.H.); fxu@sdfmu.edu.cn (F.X.); shanshan_shcn@126.com (S.S.)

# The PDF file includes:

Materials and Methods

Results

Fig. S1 Isolation and characterization of BCs derived from the small bronchi of explanted lung lobes with IPF.

Fig. S2 Single-cell transcriptomes of BCs between IPF patients and controls.

Fig. S3 The potential regulatory impact of transplanted BCs on host endogenous cells.

Fig. S4 Proliferative capacity of colonized BCs in bleomycin injured mouse lungs.

Fig. S5 The engraftment of infused cells in bleomycin-injured mouse lungs.

Fig. S6 Safety evaluation of BCs transplantation.

Fig. S7 Histological toxicity assessment of major organs in mice after transplantation.

**Other Supplementary Material for this manuscript includes the following:**

Data file S1. CNV genotypes of BCs at passage 5 and 10 compared to passage 2.

Data file S2. SNV genotypes of BCs at passage 5 and 10 compared to passage 2.

Data file S3. Lung parameters of CT scans taken before the therapy and six months postoperatively in each patient.

**Materials and Methods**

**Flow cytometric analysis**

BCs at a density of 5×10^6^ /ml were resuspended in pre-chilled PBS (with 10%FBS and 1%sodium azide). PE anti-ITGA6 antibody (Abcam, ab95703, 1:1000) was added to the cell suspension and incubated on ice for 30min. Following the incubation, BCs were washed thoroughly and resuspended in PBS for further flow cytometry analysis. Feeder cells were prepared in the same way as a negative control. Stained cells were analyzed immediately under the PE channels on Cytoflex LX (Beckman Coulter). Positive gates were set based on the negative controls (<1% of stained feeder cells were positive). The ratio of ITGA6-positive BCs in each group was quantified by CytExpert 2.4 software.

**Cell proliferation analysis**

BCs (4000 cells per well) were planted in 96-well plates and cultured feeder-free. OD values (450nm) for each well were measured by Cell Counting Kit-8 (Beyotime) at 60, 96, 132, 168 and 240 hours post seeding. Cell proliferation curves were plotted using software package GraphPad Prism 8.3.

**Clonogenicity analysis**

BCs (100 cells/cm^2^) were planted on feeder layers and the number of formed clones containing ≥10 cells were manually counted 10 days later under a Nikon Eclipse Ts2 inverted microscope. Clone formation efficiency was evaluated as number of colonies divided by number of input BCs. Clonogenicity analysis was performed by GraphPad Prism 8.3.

**PGE2 measurement**

10^6^ BCs were seeded to 100 mm dishes, and cultured for 24 h and 48 h, respectively. For PGE2 measurement, media was harvested from cultures 24 h or 48 h after incubation. Cellular debris was removed by centrifugation to collect the supernatant, and then the quantitative determination of PGE2 in cell culture supernatant was measured by ELISA using PGE2 Competitive ELISA Kit (Multi Sciences, Hangzhou, China) according to the manufacturers’ instruction.

**In vivo differentiation assays**

Cultured BCs (10^7^ cells) were resuspended in 150ul DMEM (Gibco, 11960-044) and subcutaneously injected into immunodeficient NOD-Prkdc^scid^ Il2rg^em1^/Smoc mice (M-NSG, Shanghai Model Organisms) mixed with growth factor reduced Matrigel (Corning, 356230) at a 1:1 (v/v) ratio. Mice were sacrificed at 4th week post injection, and nodule growths were collected for paraffin embedding, sectioning and subsequently immunohistochemical (IHC) analysis.

**Whole exosome sequencing**

Genomic DNA of BCs at passage 2, 5 and 10 were extracted by CTAB method and then captured using Agilent SureSelect Human All Exon v6 Kit following the manufacturer’s protocol (Agilent Technologies, USA). Briefly, about 3 micrograms of qualified genomic DNA was subjected to obtain the sheared DNA fragments (150 to 220 bp), followed by purification, adapter ligation, polymerase chain reaction (PCR), hybridization to generate the final amplified libraries. Then these libraries were sequenced on Illumina HiSeq X-10 sequencing platform and 150 bp paired-end reads were produced. The raw data were subsequently filtered with fastp (Version: 0.19.5) by removing linker sequences and low-quality reads. In detail, reads with unknown base (not AGCT) ratios >5% or average quality value below 20 in a 4-base sliding window were trimmed. Then all reads shorter than 75 bp or average quality values below 15 were eventually discarded to get the clean reads for further analysis.

Clean reads were mapped to the reference human genome (g1k_v37) utilizing BWA (version 0.7.12), followed by sorting and indexing using SAMtools (Version 1.4), and removing duplicate reads by Picard (Version 4.1.0.0). Then GATK (Version 4.1.0.0) was applied on the base quality score recalibration and SNP /Indel realignment to obtain analysis-ready BAM files, which were used as input files for variant calling. Somatic variants of BCs at passage 5, 10 compared with passage 2 were identified with Mutect2 (GATK-4.1.0.0). Several annotation databases, such as 1000 Genomes, ClinVar, the Catalogue of Somatic Mutations in Cancer (COSMIC), OMIM, and so on, were referred to analyze disease-related variants in BCs by ANNOVAR, as listed in Data file S2 and Data file S7. CNV was inferred from sequencing data using the software package CNVkit (version 0.9.5). The whole exome sequencing and bioinformatic analysis were conducted by OE Biotech Co., Ltd. (Shanghai, China).

***Single cell RNA sequencing*(*****scRNA-seq*) analysis**

Cultures of IPF and control BCs from small bronchi were harvested for *scRNA-seq* analysis. BD Rhapsody Analysis pipeline (Version: 1.8) was applied with default parameter to align reads and generate feature-barcode matrices. The reads were mapped to human genome (GENCODE version 29) utilizing STAR mapping with customized parameter from BD Rhapsody Analysis pipeline. Seurat R package (version: 3.1.5) was used for cell normalization and regression according to the UMI counts of each cell, feature filtration and mitochondrial percentage, which in turn obtained the scaled data. We have utilized the “Canonical Correlation Analysis (CCA)” method for batch effect correction. Principal component analysis (PCA) was performed based on the scaled data with top 2000 high variable genes, and top 20 principals were used for tSNE construction. Unsupervised clustering was performed to cluster BCs by the GraphClust approach. DEGs were carried out with Seurat ‘FindMarkers’ function (Wilcoxon rank sum test, P values adjusted using Bonferroni correction), and identified by satisfying these two criteria: a) the average log-transformed fold change (|avg_logFC|) > 26.5%, b) the adjusted P value (FDR) ＜ 0.05. Then, the DEGs were subjected to GO_BP enrichment. A bubble plot for the representative DEGs were generated using the R package ggplot2.

Cultures of distal airway BCs and fibroblasts from IPF patient were harvested for cell-cell interaction analysis, combined with the engrafted BCs. The single-cell sequencing data of distal airway BCs and fibroblasts were processed and analyzed as described above. CellPhoneDB, which is a novel repository of ligands, receptors and their interactions, were used to explore the interactions among distinct cells. With the aim to mimic the physiological state post-transplantation, we compared the intersection of interactions between IPF_ BCs, Control_ BCs, Fibroblasts & Distal_ BCs. These comparisons theoretically excluded the interference of non-specific interactions and explored the potential mechanisms on the therapeutic effects of basal cells (BCs). Considering such complex analyses are not easily understood, we compared the interactions between each IPF basal cell (IPF_ BCs) sample with control basal cells (Control _ BCs) as well as fibroblasts in the revised version. Since the fact that all three patients showed good therapeutic responses after cell therapy, and the intersection of the cellular interactions with Distal_ BCs and Fibroblasts in the three patients would provide insights into the potential mechanisms of autologous stem cell transplantation therapy for IPF. P-value for the cell-type-specific interaction was calculated.

**Tumorigenicity assay**

Lung cancer cells H460 (2×10^6^ cells/100μl/administration), lung interstitial cells (4×10^6^ cells/100μl/administration) and BCs (high dose of 4×10^6^ and low dose of 2×10^6^ cells/100μl/administration) were resuspended in DMEM (Gibco, 11960-044) and subcutaneously injected into M-NSG mice, respectively. Nodule size and mouse weight were monitored every 5 days. The nodule volume was calculated as 0.5× (length × width^2^). When the largest tumor volume reached 2000mm^3^ (day 30) in H460 group, 4 mice of this group were all sacrificed and tumors were removed for gross observation. The remaining groups continued to be observed until 45 days to explore the outcomes of BC nodules. At the end of this experiment, mice were euthanized and dissected. The skin tissue and subcutaneous space was carefully inspected, especially at the transplanted site. Tumor volume curves and body weight curves were plotted using GraphPad Prism 8.3.0.

**Intratracheal transplantation of BCs in mouse**

To verify the repair capability of BCs in the fibrotic lungs, we firstly constructed a pulmonary fibrosis model by intratracheal injection of bleomycin (Bleo) at a dose of 1U/kg in immunodeficient M-NSG mice. The source of bleomycin was Bleomycin from Selleck (Catalog No. S1214, Lot number S121419, Purity: 99.97%). Bleomycin-induced modeling is the most widely used and recognized method for studying the pathogenesis of IPF, with fibrosis generally considered to begin around Day 7 after induction^1^. Lung injury and fibrosis were confirmed 7 days later through micro-computed tomography (micro-CT) examination (SkyScan-1176 system, Bruker-microCT, Belgium). Immediately following randomized grouping, either saline or BC suspension (10^6^ cells/50μl/administration) were instilled intra-tracheally on established Bleo-injured model. The lung function tests were conducted once the mice’s condition is table without the signs of erratic movements or excessive activities. On day 15 and day 45 post transplantation, mice were sacrificed for tissue collection. The separated lung lobes were then subjected to fluorescent stereoscope (MZX81, Micro-shot Technology Co. Ltd., Guangzhou, China) observation, paraffin embedding, serial sectioning and subsequently HE/IHC/IF staining. The tissue morphology of lungs was observed by HE/IHC staining, and transplanted human BCs were identified with mouse anti-human nucleoli (Abcam, ab190710, 1:200). Multiple differentiation directions of colonized BCs were determined by co-staining of human nucleoli and markers of airway major cell types as mentioned above. Double-positive cells were counted to calculate the proportions of different airway cell types from in vivo differentiation of IPF-BCs.

To assess the safety of IPF-BCs infusion, we intra-tracheally injected saline or BC suspension (10^6^ cells/50μl/administration) into M-NSG mouse lungs. Gross appearance, behavioral activity or other physiological functions of mice were observed over time. After 15 or 45 days, mice were examined by chest micro-CT (SkyScan-1176 system, Bruker-microCT, Belgium), and then sacrificed to collect major organs and blood samples. Major organs (lung, heart, liver, spleen, kidney) were subjected to the gross observation and HE staining. Blood samples were used to perform serum biochemical analysis. The fresh blood samples were resting at room temperature for 2 h and centrifuging (1000× g) at 4°C for 15 min to acquire serum. Serum liver and kidney function indicators including alanine transaminase (ALT), aspartate transaminase (AST), albumin (ALB), blood urea nitrogen (BUN) and creatinine (CR) were further analyzed by a Chemray 240 clinical analyzer (Rayto, Shenzhen, China).

**Hematoxylin and eosin (HE), immunohistochemistry (IHC) and immunofluorescence (IF) staining**

BC colonies and tissue samples were fixed in 4% (w/v) paraformaldehyde, followed by directly clone staining or paraffin embedding for sectioning. Tissue sections (3μm) were then subjected to HE, IHC and IF staining after dewaxing and rehydration steps through xylene and graded alcohols.

For HE staining, deparaffinized sections were stained with hematoxylin for 5 min, washed in 1% of acid ethanol and distilled water, and then stained again with eosin for 5 min. For IHC staining, dewaxed sections were submerged in EDTA antigen retrieval buffer (pH 9.0) and microwaved for antigen repair, proceeded to incubate with primary antibodies as follows: mouse anti-CCSP antibody (MA5-17170, ThermoFisher, 1:200), mouse anti-Acetylated Tubulin (T7451, Sigma, 1:500), rabbit anti-Muc5AC (ab198294, Abcam, 1:500) and mouse anti-human nucleoli (ab190710, Abcam, 1:200). Following that, slides were reacted with secondary antibody for 2h at room temperature, and subsequently underwent color development with Vector Labs ABC kit and DAB substrate (Vector Laboratories). At last, slides were dehydrated with graded alcohol and cleared in xylene.

For IF staining, tissue sections (antigen repaired with EDTA buffer and microwave) and BC colonies were permeabilized and blocked in PBS containing 5% bovine serum albumin and 0.1% Triton X-100, and next probed with primary antibodies as noted below: rabbit anti-EPCAM (ab71916, Abcam, 1:100), mouse anti-P21 (sc-6246; Santa Cruz, 1:50), mouse anti-P16 (sc-1661; Santa Cruz, 1:50), rabbit anti-Krt5 (ab52635, Abcam, 1:200), mouse anti-p63 (ab735, Abcam, 1:200), rabbit anti-NGFR (ab52987, Abcam, 1:100), rabbit anti-Ki67 (ab16667, Abcam, 1:100), mouse anti-Krt8 (NBP1-48281, Novus Bio, 1:50), mouse anti-human nucleoli (ab190710, Abcam, 1:500), rabbit anti-CCSP antibody (10490-1-AP, Proteintech, 1:200), rabbit anti-Foxj1 (NBP1-87928, Novus Bio, 1:800), rabbit anti-Muc5AC (ab198294, Abcam, 1:500), rabbit anti-Fibronectin (ab268020, Abcam, 1:500), mouse anti-Vimentin (ab8978, Abcam, 1:500), mouse anti-α-SMA (A2547, Sigma, 1:400) and rabbit anti-collagen I (#72026, Cell Signaling Technology, 1:100). Then, proper Alexa Fluor 488 or 594 conjugated secondary antibodies (ab150077, ab150113, ab150080, ab150116, Abcam, 1:1000) were used for immunofluorescence labeling.

The antibodies involved in our study have been conducted validation for the experimental applications by the suppliers, adhering to knockout validation, which is one of the five conceptual pillars proposed by the International Working Group on Antibody Validation (IWGAV). Additionally, the effectiveness of these antibodies has been demonstrated in multiple published studies. In our work, the antibodies accurately localize within the cells, show distinct differences in protein expression between groups, and exhibit strong specificity, thereby ensuring the appropriateness and accuracy of their application.

All the results of HE, IHC and IF staining were visualized and photographed by Olympus IX53 microscope (Tokyo, Japan).

**Results**

**Isolation and characterization of BCs derived from the small bronchi of explanted lung lobes with IPF**

We then collected small bronchi-derived BCs from explanted lungs with IPF via bronchial epithelial brushing near the opening of the left or right basal segment (fifth-generation bronchi), followed by scaled expansion *in vitro* (Figure S1A). Both BCs isolated from bronchoscopic brushings and thus cultured BCs clones displayed a non-senescence phenotype (Figure S1B-C). Using this approach, non-senescent BCs were successfully obtained in isolated lung tissues of IPF, suggesting a theoretical potential for cellular therapy involving BCs for the treatment of IPF.

**Transcriptomic signatures of BCs in IPF**

To further evaluate the cultured BCs, their transcriptomic signatures were identified by *scRNA-seq* analysis. 28373 BCs from three IPF (14520 BCs) and three control (13853 BCs) samples were profiled (Figure S2A). Good agreement was found across various samples or different groups due to their high overlap of expression profiles with each other (Figure S2B).

Two major BCs subpopulations, ‘Cluster A’ and ‘Cluster B’, were further defined based on reported biomarkers^2^ (Figure S2D). The top Gene Ontology Biological Process (GO_BP) terms (Figure S2E) were identified in each subpopulation. Genes related to inflammation (e.g., S100A8/A9, CXCL17, PI3, SLPI, KRT6B, KRT6C, KRT16, CSTB) ^3-6^, keratinocyte differentiation (e.g., KRT4, KRT13, SPRR family, IVL) ^7-10^, and mucus-secretion (e.g., AGR2) ^11^ were significantly enriched in cluster B, while the proliferation markers (e.g., KI67, PCNA, TOP2A), anti-oxidant and anti-apoptotic markers (e.g., MT2A, MT1E) ^12^, and classical BCs markers (e.g., ITGA6) were high expressed in cluster A. Similar distributions of BCs subpopulations were detected between control and IPF groups(Figure S2C), showing cluster A cells in the vast majority of the BCs population (95-97% in patient-derived cultures versus 94-96% in controls). Despite a small portion of cluster B cells (approximately 4% of total) existing in IPF samples, this was attributed to a background ratio when compared with controls. Moreover, a natural existence of cluster B cells, which accounted for less than 10% of the total BCs, appeared to have little impact on overall characteristics and differentiation outcomes of BCs when compared IPF and control groups ^2^.

The senescence scores of each sample were calculated based on three sets of aging-related genes in GO or GSEA database. Both IPF BCs and control BCs exhibited low senescence score values, with no statistically significant difference (Figure S2F). We additionally assessed the level of stemness in each sample within our *scRNA-seq* data (Figure S2G) The results indicated that the transcriptional diversity associated with stem cell properties showed no distinction between IPF group and controls.

The potential regulatory impact of transplanted BCs on host endogenous cells should be taken into consideration by analysis of *scRNA-seq* data. Investigating the interaction between transplanted BCs and distal airway BCs or fibroblasts (the initiator and key effector cells in the fibrosis process), may contribute to a broader discourse on the promising therapeutic effects of BCs. With the aim to mimic the physiological state post-transplantation, we compared the intersection of interactions between IPF_BCs, Control _ BCs, Fibroblasts & Distal_ BCs. These comparisons theoretically excluded the interference of non-specific interactions and explored the potential mechanisms on the therapeutic effects of basal cells (BCs). Considering such complex analyses are not easily understood, we compared the interactions between each IPF basal cell (IPF_BCs) sample with control basal cells (Control _ BCs) as well as fibroblasts in the revised version. Since the fact that all three patients showed good therapeutic responses after cell therapy, and the intersection of the cellular interactions with Distal_BCs and FIBROBLASTS in the three patients would provide insights into the potential mechanisms of autologous stem cell transplantation therapy for IPF (Figure S3A-B). Our *scRNA-seq* data, in conjunction with a thorough literature review, suggest that these interactions are important for promoting tissue repair, and exerting anti-fibrotic effects through several mechanisms. Following airway injury, the engrafted BCs isolated from small bronchi transmit Wnt, FGF1 and EGF PGE2 signaling to the distal airway BCs, which coordinate *in vivo* to promote epithelial repair^13-15^. PGE2 and FGF family members (such as FGF1/2/9) are widely recognized for their role to attenuate fibrosis by decreasing fibroblast differentiation and stress fiber formation in the lung ^14,16,17^. In particular, PGE2 emerged as a pivotal factor mediating therapeutic efficacy in IPF treatment^18^. Our study revealed that some BCs and their terminally differentiated cells expressed PGE synthases (PTGES) (Figure S3C-D), and could further secret PGE2 (Figure S3E). PGE2, as a common predicted interaction target of cultured basal cells (BCs) with distal basal cells (Distal BCs) and fibroblasts, holds significant research importance. Overall, it is plausible to hypothesize that the transplanted BCs may mediate anti-fibrotic effect, thereby demonstrating their therapeutic potential for IPF treatment.

**Safety assessment of BCs transplantation**

Given the clinical promise of BCs for IPF treatment, we performed preclinical safety assessment to evaluate major organ toxicity and tumorigenicity, thereby facilitating the transition to clinical application.

IPF-BCs at 5×10^7^/kg dose level (25-fold higher than clinical application) were instilled intratracheally into M-NSG mouse lungs, followed by a 15-day short-term and 45-day long-term observation (Figure S6A). No overt abnormalities were observed with regard to gross appearance, behavioral activity or other physiological functions were seen in all treated mice during the monitoring period. The potential acute or chronic toxicities of BCs towards major organs were examined by histology, imaging, and blood biochemistry. At both 15 days and 45 days post-transplantation, histological analysis of major organs revealed no appreciable signs of toxicity such as tissue inflammation, degeneration, necrosis, or regeneration in BCs-treated mice, compared to PBS-treated (Figure S6B, Figure S7). Similarly, chest micro-CT imaging verified that BCs treatment never induced pulmonary inflammation, tumorigenesis, focal adhesions or pleural effusion regardless of short-term or long-term follow-up post infusion (fig. S6B). In addition, BCs transplantation did not affect liver and renal functions (liver functional markers: ALT, AST and ALB; renal functional markers: BUN, CREA), as demonstrated in Figure S6C.

To proceed with tumorigenicity assessment, patient-derived BCs at overdosages (50-fold and 100-fold higher than clinical application) were used to perform *in vivo* tumorigenic assay, in parallel with a positive control of human lung cancer cells H460 and a negative control of primary human lung interstitial cells (Figure S6D). No tumorigenic risk was verified for BCs groups due to the spontaneous regression of BCs nodules after 45 days (Figure S6D). Volume change curves of nodules varying from different cell types were shown in Figure S6E. Body weight of BCs group rose steadily after operation consistent with the range of negative controls, while a slightly decrease were presented in positive controls compared to the initial weight (Figure S6F).

Based on these comprehensive results, the cultures of patient-derived BCs have demonstrated a high degree of feasibility for translational application.


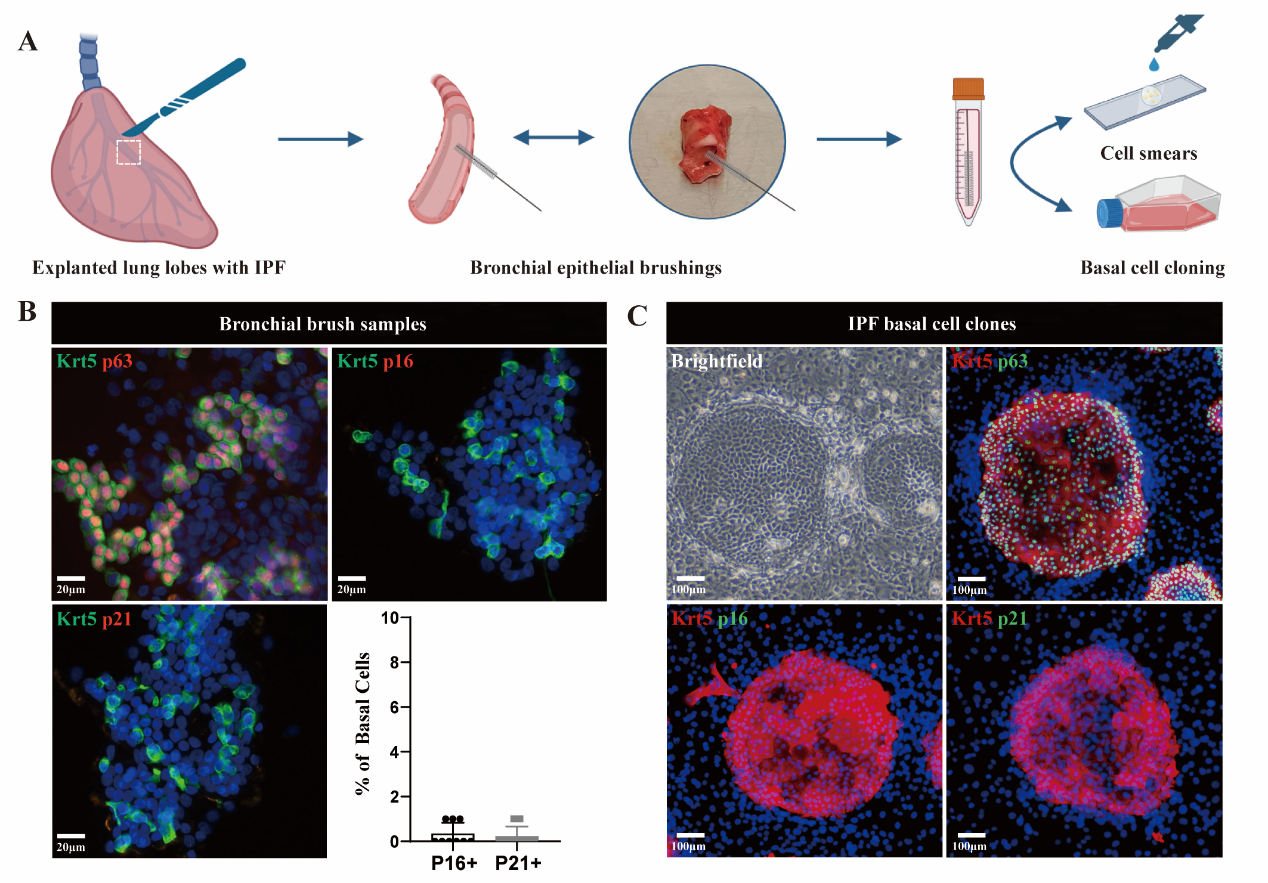


**Fig. S1 Isolation and characterization of BCs derived from the small bronchi of explanted lung lobes with IPF. (A)** A schematic diagram illustrating the process of obtaining BCs from the small bronchi of explanted lung lobes with IPF. **(B-C)** Identification of senescence markers p16 and p21 in BCs isolated from bronchoscopic brushings (B) and thus cultured BCs clones (C) by immunofluorescence staining.


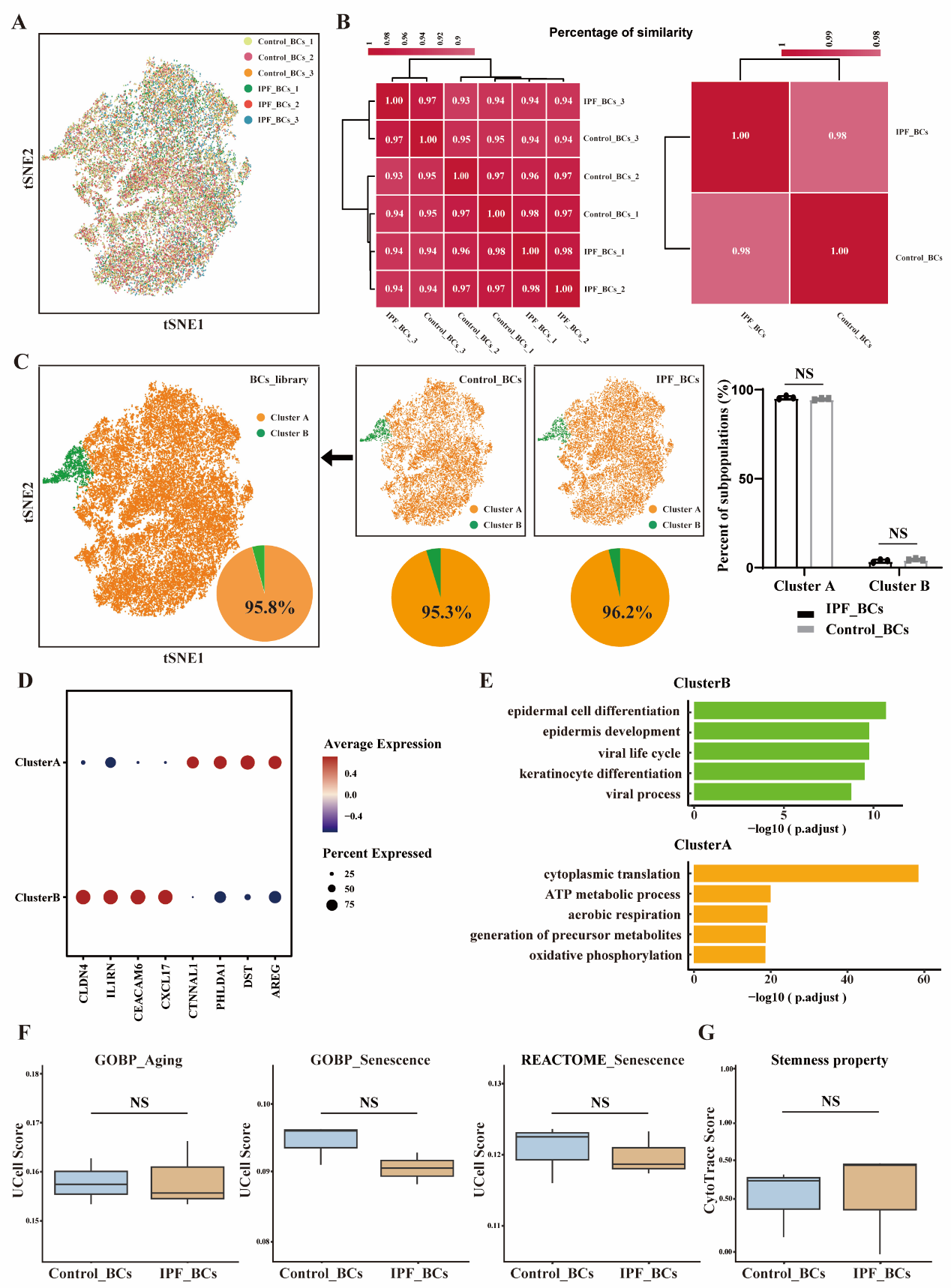


**Fig. S2 Single-cell transcriptomes of BCs between IPF patients and controls. (A)** Aggregate tSNE map of BCs from three IPF and three control cultures, colored by samples. **(B)** Consistency analysis among different samples and groups, with numerical values representing the percentage of similarity, which is measured by Pearson correlation. **(C)** Aggregate tSNE map of BCs from IPF and control groups, colored by subpopulations (Cluster A, yellow; Cluster B, green). Pie charts indicate the distribution of two subpopulations in each group (Cluster A, yellow; Cluster B, green). Histograms represent the fractional contributions of typical and responsive BCs across IPF and control groups. **(D)** Dot plot depicting the expression levels of the cell marker genes in Cluster A and Cluster B. Dot color: average expression levels, dot size: percent expressed the corresponding BC markers. **(E)** Histogram displaying the top 5 enriched GO_BP terms determined by GO Analysis of the DEGs in Cluster A and Cluster B (Cluster A, yellow; Cluster B, green). **(F)** The senescence scores of both IPF BCs and control BCs based on three sets of aging-related genes in GO or GSEA database(N=3). **(G)** The stemness level of both IPF BCs and control BCs by analysis of *scRNA-seq* data set. Data are presented as mean ± SD. NS denotes no statistical significance (P > 0.05). An unpaired two-tailed Student’s t-test was used for panel C, and the Wilcoxon signed-rank test was used for panels D and E. The number of patients who received autologous BCs transplantation in this study was 3.


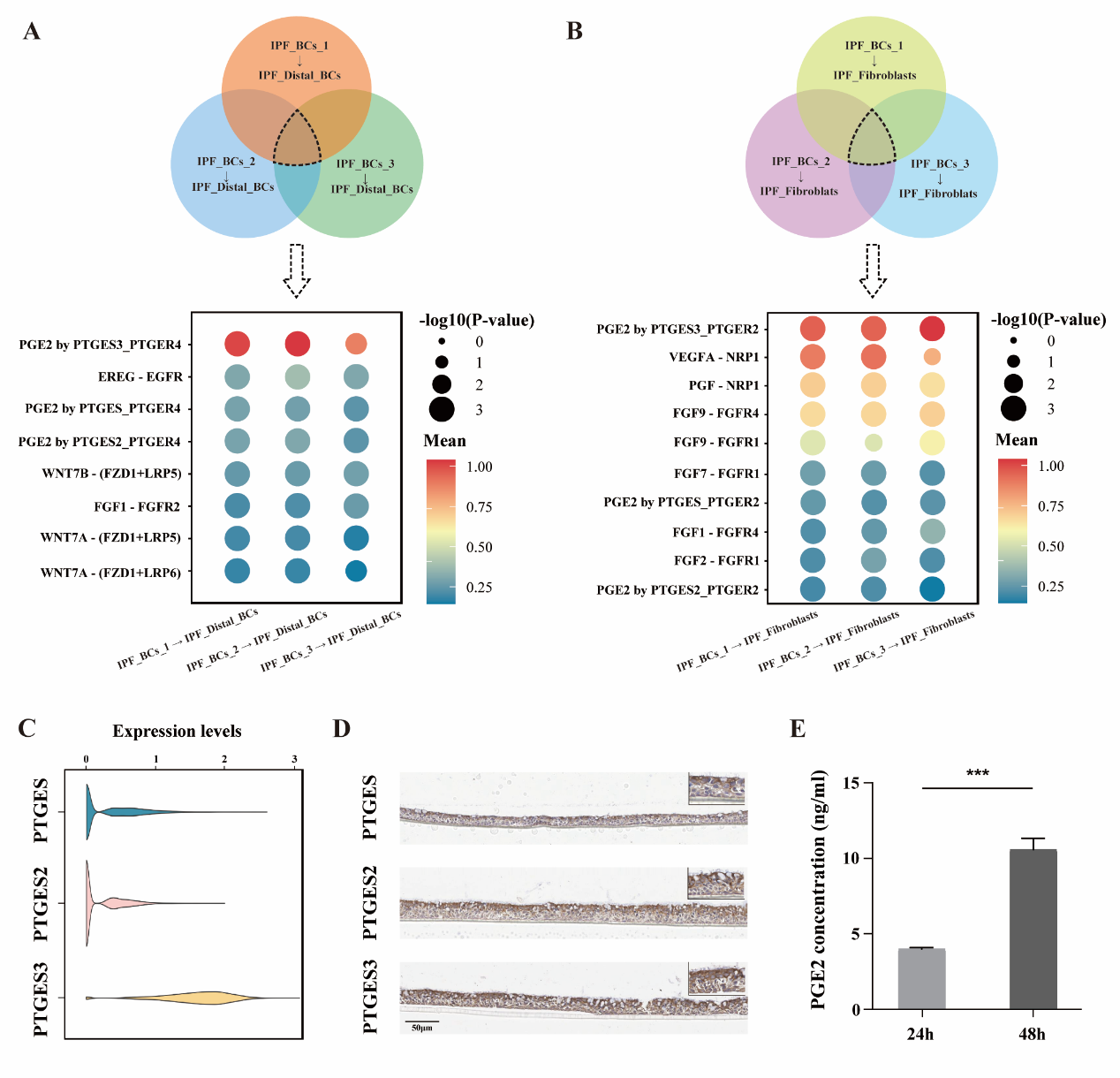


**Fig. S3** **The potential regulatory impact of transplanted BCs on host endogenous cells. (A-B)** The intersections of the interaction in three IPF patients between BCs from distal and proximal airways (A) or intersections of the interaction in three IPF patients between proximal BCs and fibroblast (B) and the downstream enrichment results of these intersection gene lists. **(C)** Violin diagrams demonstrate the expression levels of PTGE, PTGE2 and PTGE3 in BCs from IPF group. **(D)** The experimental system at air-liquid interface elucidates the expression patterns of PTGES, PTGE2 and PTGE3 in BCs and their well-differentiated counterparts. **(E)** PGE2 concentration of the supernatants of IPF BCs cultures after incubation 24h and 48h. The amount of PGE2 is significantly higher in supernatant after 48h culture in comparison with 24h culture, indicating that IPF BCs could secrete PGE2. Data are presented as mean ± SD. N=3, ***indicated P<0.001, An unpaired two-tailed Student’s t-test was used for panel E.


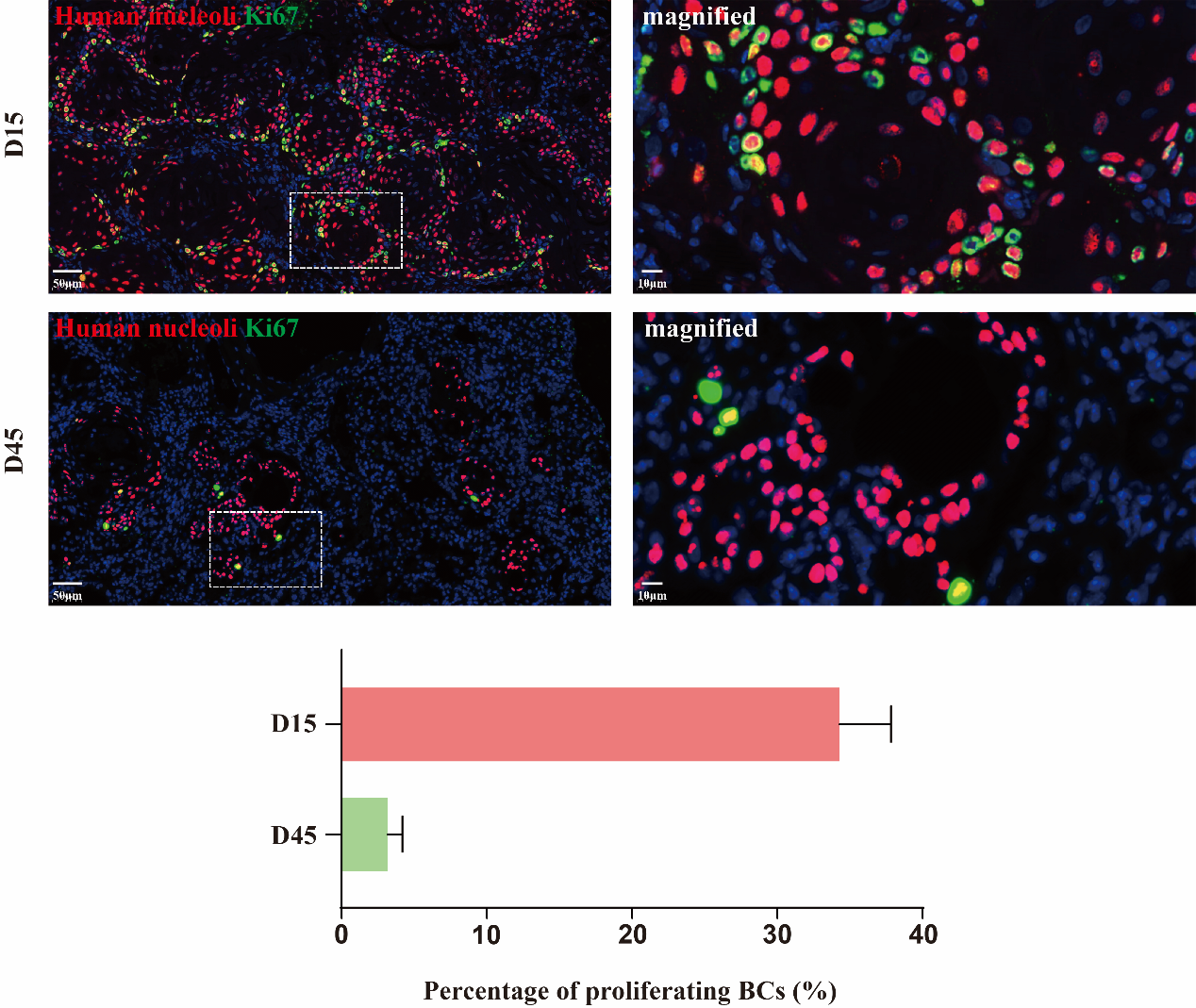


**Fig. S4. Proliferative capacity of colonized BCs in bleomycin injured mouse lungs.** Representative fluorescence micrographs demonstrate the proliferation capacity of engrafted BCs at day 15 and day 45 by staining the proliferative marker Ki67. Summary statistics of Ki67-positive cells are provided in bottom panel. Human nucleoli, red; Ki67, green. 6 fields from 3 mice per group were analyzed.


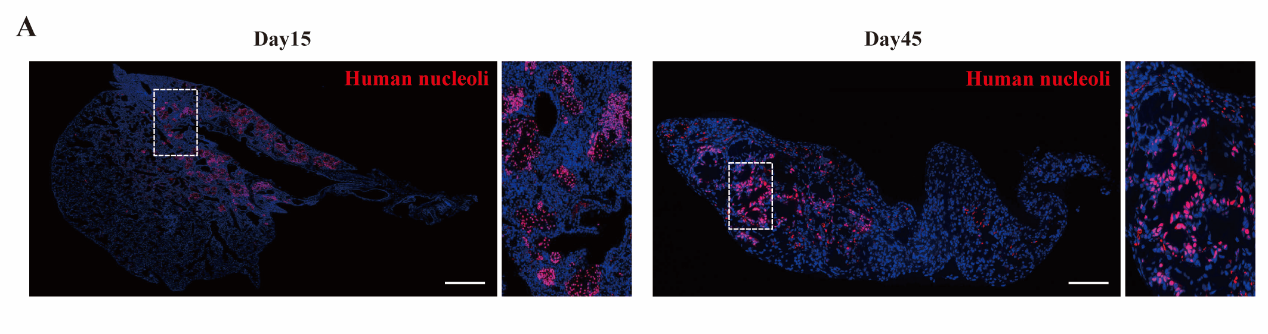


**Fig. S5 The engraftment of infused cells in bleomycin-injured mouse lungs. Scale bar 100μm.**


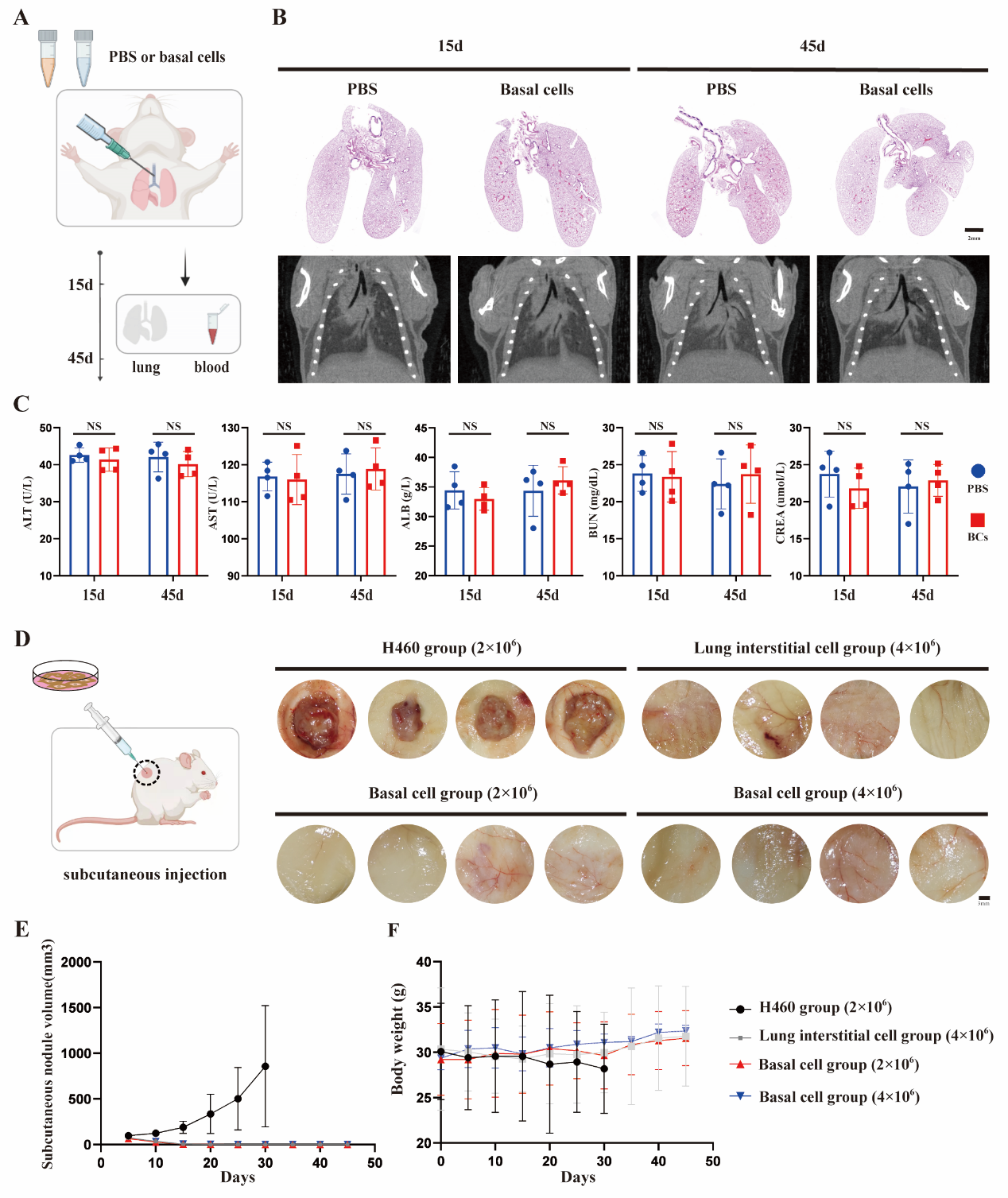


**Fig. S6 Safety evaluation of BCs transplantation. (A)** Study flowchart of an intratracheal transplantation trail to assess safety of IPF-BCs in M-NSG. **(B)** The histologic and radiographic analyses of mouse lungs at day 15 (short-term) and day 45 (long-term) post transplantation. Scale bar, 2mm. **(C)** Values of liver functional indicators ALT, AST and ALB, as well as renal functional indicators BUN and CREA in experimental mice at day 15 and day 45 after transplantation. **(D)** Representative images of subcutaneous conditions at the transplanted site until the end of the experiments. Transplants: tumor cells H460 (d30, positive control), interstitial cells (d45, negative control), basal cells (d45, high dose and low dose). Scale bar, 3mm. **(E)** Curves of volume change over time for subcutaneous nodules varying from different cell types. **(F)** Curves of body weight change over time for mice in different groups mentioned above. n=4 in each group. Results are represented as mean ± SD, NS indicated no statistical significance determined by one-way ANOVA (P＞0.05).


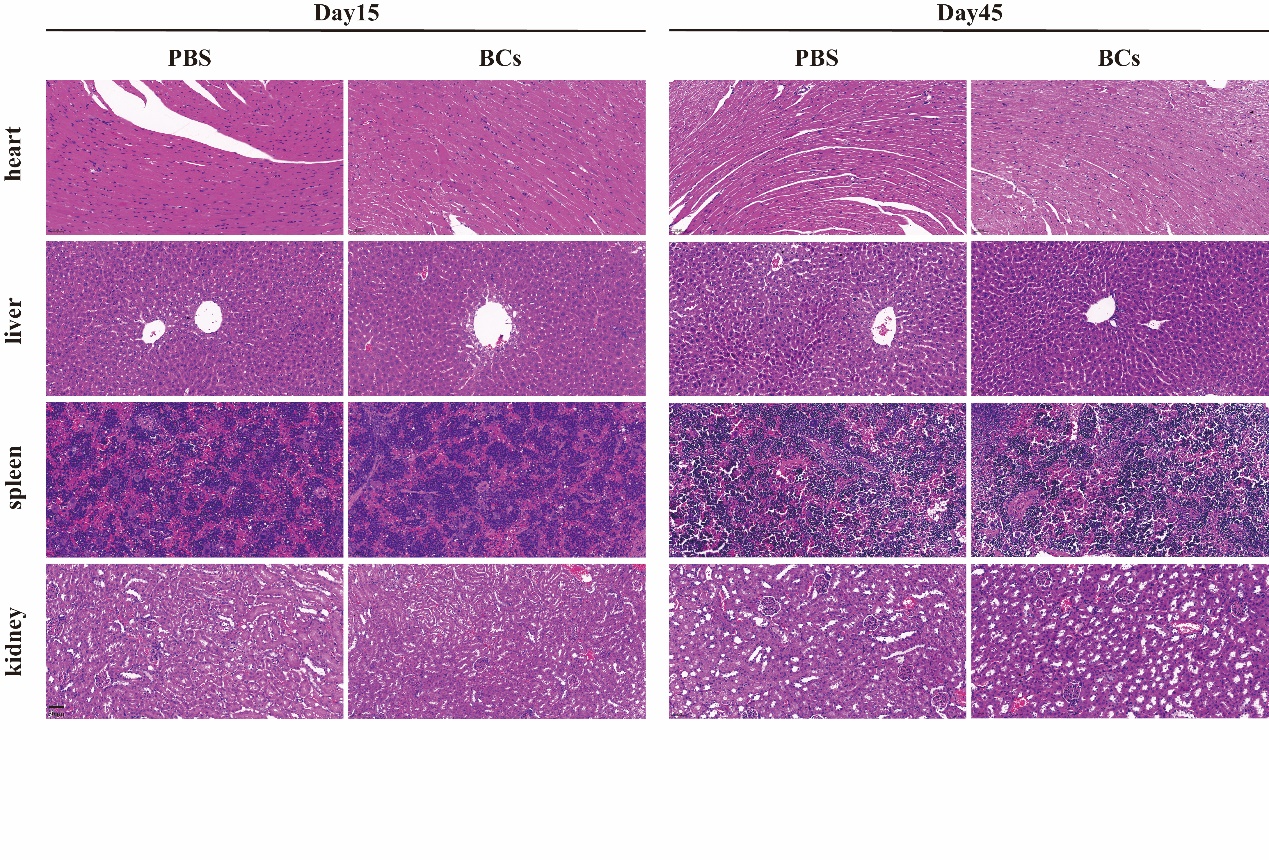


**Fig. S7 Histological toxicity assessment of major organs in mice after transplantation.** Histologic images of mouse major organs at day 15 (short-term) and day 45 (long-term) post intratracheal transplantation of BCs are shown in the picture. n=4 in each group. Scale bar, 50 μm.

**References**

1. Kolb P, Upagupta C, Vierhout M, et al. The importance of interventional timing in the bleomycin model of pulmonary fibrosis. *Eur Respir J* 2020; **55**(6).

2. Wang S, Rao W, Hoffman A, et al. Cloning a profibrotic stem cell variant in idiopathic pulmonary fibrosis. *Sci Transl Med* 2023; **15**(693): eabp9528.

3. Pruenster M, Vogl T, Roth J, Sperandio M. S100A8/A9: From basic science to clinical application. *Pharmacol Ther* 2016; **167**: 120-31.

4. Choreño-Parra JA, Thirunavukkarasu S, Zúñiga J, Khader SA. The protective and pathogenic roles of CXCL17 in human health and disease: Potential in respiratory medicine. *Cytokine Growth Factor Rev* 2020; **53**: 53-62.

5. Brunner PM, Suárez-Fariñas M, He H, et al. The atopic dermatitis blood signature is characterized by increases in inflammatory and cardiovascular risk proteins. *Sci Rep* 2017; **7**(1): 8707.

6. Rojahn TB, Vorstandlechner V, Krausgruber T, et al. Single-cell transcriptomics combined with interstitial fluid proteomics defines cell type-specific immune regulation in atopic dermatitis. *J Allergy Clin Immunol* 2020; **146**(5): 1056-69.

7. Plasschaert LW, Žilionis R, Choo-Wing R, et al. A single-cell atlas of the airway epithelium reveals the CFTR-rich pulmonary ionocyte. *Nature* 2018; **560**(7718): 377-81.

8. Hong Y, Shan S, Gu Y, et al. Malfunction of airway basal stem cells plays a crucial role in pathophysiology of tracheobronchopathia osteoplastica. *Nat Commun* 2022; **13**(1): 1309.

9. Rao W, Wang S, Duleba M, et al. Regenerative Metaplastic Clones in COPD Lung Drive Inflammation and Fibrosis. *Cell* 2020; **181**(4): 848-64.e18.

10. Goldfarbmuren KC, Jackson ND, Sajuthi SP, et al. Dissecting the cellular specificity of smoking effects and reconstructing lineages in the human airway epithelium. *Nat Commun* 2020; **11**(1): 2485.

11. Jach D, Cheng Y, Prica F, Dumartin L, Crnogorac-Jurcevic T. From development to cancer - an ever-increasing role of AGR2. *Am J Cancer Res* 2021; **11**(11): 5249-62.

12. Si M, Lang J. The roles of metallothioneins in carcinogenesis. *J Hematol Oncol* 2018; **11**(1): 107.

13. Chakraborty A, Mastalerz M, Ansari M, Schiller HB, Staab-Weijnitz CA. Emerging Roles of Airway Epithelial Cells in Idiopathic Pulmonary Fibrosis. *Cells* 2022; **11**(6).

14. Shimbori C, Bellaye PS, Xia J, et al. Fibroblast growth factor-1 attenuates TGF-β1-induced lung fibrosis. *J Pathol* 2016; **240**(2): 197-210.

15. Mora AL, Rojas M, Pardo A, Selman M. Emerging therapies for idiopathic pulmonary fibrosis, a progressive age-related disease. *Nat Rev Drug Discov* 2017; **16**(11): 755-72.

16. Koo HY, El-Baz LM, House S, et al. Fibroblast growth factor 2 decreases bleomycin-induced pulmonary fibrosis and inhibits fibroblast collagen production and myofibroblast differentiation. *J Pathol* 2018; **246**(1): 54-66.

17. Joannes A, Brayer S, Besnard V, et al. FGF9 and FGF18 in idiopathic pulmonary fibrosis promote survival and migration and inhibit myofibroblast differentiation of human lung fibroblasts in vitro. *Am J Physiol Lung Cell Mol Physiol* 2016; **310**(7): L615-29.

18. Khan P, Fytianos K, Blumer S, et al. Basal-Like Cell-Conditioned Medium Exerts Anti-Fibrotic Effects In Vitro and In Vivo. *Front Bioeng Biotechnol* 2022; **10**: 844119.
