# Supplementary figures and images for: Epithelial stem cells from human small bronchi offer a potential for therapy of idiopathic pulmonary fibrosis
Source: eBioMedicine. 2025 Jan 2;112:105538. doi: 10.1016/j.ebiom.2024.105538 (PMC11754162; doi:10.1016/j.ebiom.2024.105538)

**Graphical Abstract**

**
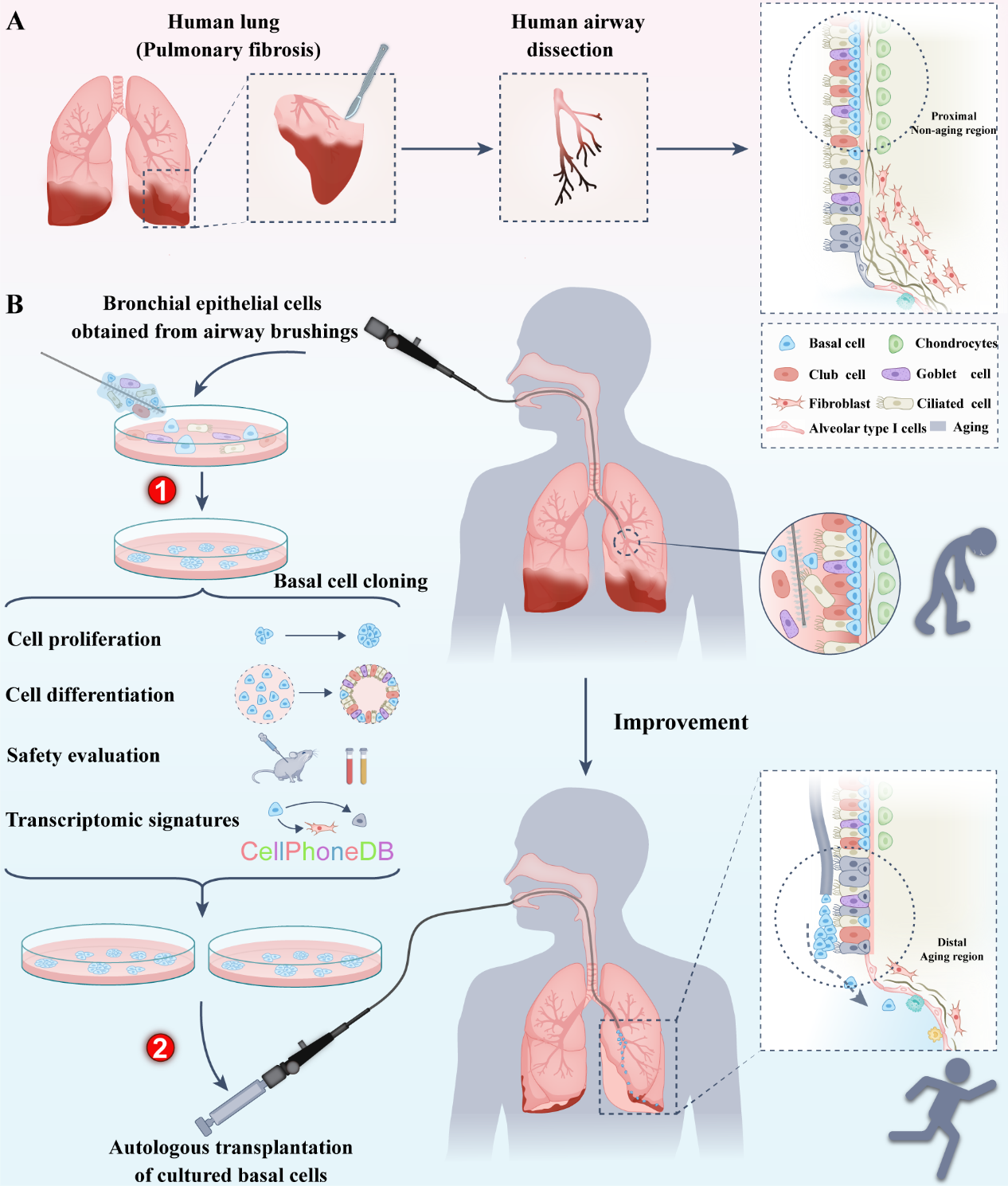
**

Supplement: Graphical Abstract [file mmc5.docx]
